# Associations of lipid measures with total occlusion in patients with established coronary artery disease: a cross-sectional study

**DOI:** 10.1186/s12944-022-01733-8

**Published:** 2022-11-11

**Authors:** Tianyu Li, Deshan Yuan, Peizhi Wang, Sida Jia, Ce Zhang, Pei Zhu, Ying Song, Xiaofang Tang, Xueyan Zhao, Zhan Gao, Yuejin Yang, Runlin Gao, Bo Xu, Jinqing Yuan

**Affiliations:** 1 National Clinical Research Center for Cardiovascular Diseases, State Key Laboratory of Cardiovascular Disease, National Center for Cardiovascular Diseases, Fuwai Hospital, Chinese Academy of Medical Sciences and Peking Union Medical College, 167, North Lishi Road, Xicheng District, Beijing, 100037 China; 2 Department of Cardiology, National Center for Cardiovascular Diseases, Fuwai Hospital, Chinese Academy of Medical Sciences and Peking Union Medical College, 167, North Lishi Road, Xicheng District, Beijing, 100037 China; 3 Catheterization Laboratories, National Center for Cardiovascular Diseases, Fuwai Hospital, Chinese Academy of Medical Sciences and Peking Union Medical College, 167, North Lishi Road, Xicheng District, Beijing, 100037 China

**Keywords:** Lipids, Lipoproteins, Coronary occlusion, Coronary artery disease

## Abstract

**Background:**

Total occlusion is the most severe coronary lesion, indicating heavy ischemic burden and poor prognosis. The lipid profile is central to the development of atherosclerotic coronary lesions. Evidence on the optimal lipid measure to be monitored and managed in patients with established coronary artery disease (CAD) is inconclusive.

**Methods:**

Total cholesterol (TC), total triglyceride (TG), low-density lipoprotein cholesterol (LDL-c), nonhigh-density lipoprotein cholesterol (non-HDL-c), lipoprotein (a) [Lp(a)], apolipoprotein B (apoB), non-HDL-c/HDL-c, and apoB/apoA-1 were analyzed in quintiles and as continuous variables. The associations of lipid measures with total occlusion were tested using logistic regression models, visualized with restricted cubic splines, and compared by areas under the receiver operating characteristic curves (AUROC). Discordance analysis was performed when apoB/apoA-1 and non-HDL-c/HDL-c were not in concordance.

**Results:**

The prospective cohort study included 10,003 patients (mean age: 58 years; women: 22.96%), with 1879 patients having total occlusion. The risks of total occlusion significantly increased with quintiles of Lp(a), non-HDL-c/HDL-c, and apoB/apoA-1 (all *p* for trend < 0.001). TG had no association with total occlusion. Restricted cubic splines indicate significant positive linear relations between the two ratios and total occlusion [odds ratio per 1-standard deviation increase (95% confidence interval): non-HDL-c/HDL-c: 1.135 (1.095–1.176), *p* < 0.001; apoB/apoA-1: 2.590 (2.049–3.274), *p* < 0.001]. The AUROCs of apoB/apoA-1 and non-HDL-c/HDL-c were significantly greater than those of single lipid measures. Elevation in the apoB/apoA-1 tertile significantly increased the risk of total occlusion at a given non-HDL-c/HDL-c tertile but not vice versa.

**Conclusion:**

ApoB/apoA-1 confers better predictive power for total occlusion than non-HDL-c/HDL-c and single lipid measures in established CAD patients.

**Supplementary Information:**

The online version contains supplementary material available at 10.1186/s12944-022-01733-8.

## BACKGROUND

Coronary artery disease (CAD) refers to myocardial ischemia due to progressive atherosclerotic lesions narrowing the artery lumen. Total occlusion, caused by acute plaque rupture or atherosclerotic plaque progression, represents the most advanced lesions that completely interrupt coronary blood flow. Acute total occlusion often leads to acute myocardial infarction (AMI) [1], while chronic total occlusion is also associated with adverse prognosis [2, 3]. Treatment of total occlusion is a challenging issue; hence, the prevention of atherosclerotic lesions from developing to total occlusion is crucial in the management of CAD.

Easily measurable blood markers for the prediction of total occlusion in CAD patients have always been a topic of interest. Many hematological, biochemical and inflammatory parameters have been examined to predict acute or chronic total occlusion [4–6]. However, evidence concerning the association of lipid profiles with total occlusion is limited. Lipids, lipoprotein, and apolipoprotein are core factors in the initiation and progression of atherosclerosis. Previous studies have discussed the predictive power of single lipid measures and lipid ratios for the severity of coronary lesions and the risk of fatal myocardial infarction in patients without CAD [7, 8]. We intended to investigate the associations of different lipid measures with total occlusion in a large cohort of patients with established CAD and to determine which measures are more relevant to total occlusion and should be noted in the clinical management of CAD.

## Methods

### Study design, setting, and participants

The study cohort comprised 10,724 consecutive patients undergoing percutaneous coronary intervention (PCI) from January 2013 to December 2013 at Fuwai Hospital, Chinese Academy of Medical Sciences, Beijing, China. Demographic, clinical and medication information was extracted from the electronic medical records. The cross-sectional study generated post hoc analysis of data from the cohort above and compared total cholesterol (TC), total triglyceride (TG), low-density lipoprotein cholesterol (LDL-c), nonhigh-density lipoprotein cholesterol (non-HDL-c), lipoprotein (a) [Lp(a)], apolipoprotein B (apoB), non-HDL-c/HDL-c, and apoB/apoA-1 in the prediction of total occlusion. Patients with previous coronary artery bypass grafting and missing data on lipid measures were excluded.

### Blood sampling and laboratory analysis

Venous blood samples were collected after fasting for at least 12 h and assayed within 24 h of admission. Lipid measures were analyzed using an automatic biochemistry analyzer (Hitachi 7150, Tokyo, Japan). In detail, apoB, apoA-1, and Lp(a) were measured by an immunoturbidimetric method. TC, TG, LDL-c, and HDL-c were measured by an enzymatic method. Non-HDL-c was calculated as TC minus HDL-c. Fasting glucose was assayed by an enzymatic hexokinase method. HbA1c was assayed using a Tosoh Automated Glycohemoglobin Analyzer (HLC-723G8, Tokyo, Japan).

### Definitions

Total occlusion was defined as occlusion of the coronary artery with thrombolysis in myocardial infarction (TIMI) grade 0 flow in the distal segment of the completely occluded vessel due to atherosclerosis. A body mass index ≥ 28 kg/m^2^ was considered to indicate obesity [9]. Diabetes was defined as glycated hemoglobin (HbA1c) > 6.5% or self-reported diabetes. Hypertension was defined as a mean blood pressure ≥ 140/90 mmHg or self-reported hypertension. Dyslipidemia was diagnosed when at least one of the following criteria was met: TC ≥ 6.22 mmol/L; TG ≥ 2.26 mmol/L; LDL-c ≥ 4.14 mmol/L; HDL-c < 1.04 mmol/L; or self-reported lipid-lowering medication use [10].

### Statistical analysis

Details on the estimation of the sample size can be found in the Supplemental Methods in Additional File. Lipid measures were analyzed in quintiles and as continuous variables. The distributions of lipid measures are depicted in histograms. Correlation between each pair of lipid measures was assessed using scatterplot (data not shown) and Spearman rank correlation analysis without adjustment. Baseline characteristics across quintiles of each lipid measure and between patients with or without occlusion were compared using Kruskal‒Wallis tests, χ2 tests for trend, Mann‒Whitney U test, or χ2 tests, as appropriate. Categorical variables are expressed as numbers (percentages). Continuous variables are expressed as the median [interquartile range].

The associations of lipid measures with total occlusion were examined using logistic regression models by estimating odds ratios (ORs) and 95% confidence intervals (CIs). Model 1 was unadjusted; Model 2 was adjusted for sex and age; Model 3 was adjusted for sex, age, body mass index, hypertension, diabetes, previous MI, previous PCI, smoking history, and admission presentation. The relations between lipid measure levels and total occlusion were visualized with restricted cubic splines with 4 knots, adjusting for all variables in Model 3. The median of each measure was set as the reference. The receiver operating characteristic curve (ROC) was used to evaluate the predictive power of each lipid measure (continuous) for total occlusion. The areas under the ROC (AUROCs) were compared using a nonparametric approach [11] and the integrated discrimination improvement measure. Subgroup analysis was performed according to four prespecified variables of interest—sex, age, diabetes status, and admission presentation—to calculate the OR (95% CI) and AUROC of each lipid measure (continuous).

A discordance analysis was further performed to quantify the associations of apoB/apoA-1 and non-HDL-c/HDL-c with total occlusion when the two ratios were not in concordance. ApoB/apoA-1 and non-HDL-c/HDL-c were categorized into tertiles (low, middle, high). Concordance was defined as apoB/apoA-1, and non-HDL-c/HDL-c levels were in the same tertile. Discordance was defined as apoB/apoA-1 and non-HDL-c/HDL-c in different tertiles. Baseline characteristics and ORs (95% CIs) across the nine concordance/discordance groups were analyzed as described above.

Since the lipid profile can be influenced by acute stress, all analyses were repeated in patients with acute coronary syndrome (ACS) as a sensitivity analysis to assess the robustness of our findings.

Statistical analyses were conducted with R version 3.6.3 (R Core Team 2020, Vienna, Austria. www.R-project.org). Figures were created by GraphPad Prism version 8.0.2 (GraphPad Software, San Diego, California, USA, www.graphpad.com). Two-tailed *P* values of < 0.05 were considered statistically significant.

## Results

### Study population and baseline characteristics

A total of 10,003 CAD patients were included in this analysis after excluding 437 patients with previous coronary artery bypass grafting and 284 patients with missing data for apoB and apoA-1. The mean age of the study population was 58 years (range: 18–91), and 22.96% were women. Supplemental Fig. 1 in Additional file shows the distribution of each lipid measure. TC, LDL-c, non-HDL-c, and apoB were strongly positively correlated with each other. Non-HDL-c/HDL-c strongly positively correlated with apoB/apoA-1. TG had a weak to medium correlation with other measures, while Lp(a) did not correlate with other measures (Table 1).

**Table 1:** Spearman correlation coefficients r between each pair of lipid measures

	**TC**	**TG**	**LDL-c**	**Non-HDL-c**	**Lp(a)**	**ApoB**	**Non-HDL-c** **/HDL-c**
TG	0.386						
LDL-c	0.922	0.245					
Non-HDL-c	0.954	0.491	0.922				
Lp(a)	0.124	-0.088	0.165	0.113			
ApoB	0.860	0.439	0.859	0.892	0.200		
Non-HDL-c /HDL-c	0.551	0.572	0.601	0.757	0.046	0.656	
ApoB/apoA-1	0.554	0.397	0.661	0.704	0.180	0.815	0.816

r ≤ 0.2: no correlation; 0.2 < r ≤ 0.4: weak correlation; 0.4 < r ≤ 0.6: medium correlation; 0.6 < r ≤ 0.8: high correlation; r > 0.8: strong correlation. LDL-c, low-density lipoprotein cholesterol; HDL-c, high-density lipoprotein cholesterol; lp(a), lipoprotein (a); apo, apolipoprotein

The proportions of women increased with quintiles of all single lipid measures but decreased with quintiles of the two lipid ratios. Patients in higher Lp(a) quintiles were older and had lower body mass index, whereas patients in higher quintiles of other measures were younger and more likely to be obese. Higher quintiles of each lipid measure had more patients presenting with AMI. The proportion of smokers increased with quintiles of TG and lipid ratios and decreased with quintiles of TC, LDL-c, and Lp(a) but had no significant difference among quintiles of apoB and non-HDL-c. The proportion of diabetic patients increased with quintiles of TC and lipid ratios but was comparable among quintiles of other measures. Insulin use did not differ significantly among quintiles of each lipid measure. Patients in higher quintiles of each lipid measure had less use of oral antidiabetic agents and more elevated fasting glucose and HbA1c levels. Patients with higher levels of each lipid measure were less likely to have a history of MI, stroke, or PCI. Other characteristics were not significantly different among quintiles of each lipid measure (see Supplemental Tables 1,2,3,4,5,6,7,8 in Additional File).

Total occlusion was detected by coronary angiography in 1879 patients. Patients with total occlusion were more likely to be men, younger and obese; they were also more likely to present with AMI and have a history of smoking, MI, and PCI. Higher fasting glucose and HbA1c levels, lower estimated glomerular filtration rates and lower left ventricular ejection fractions were observed in the total occlusion group (Table 2). The statistically significant but slight differences in lipid measures between patients with and without total occlusion were probably due to Type I error in large samples, which may yield no clinical significance.

**Table 2:** Baseline characteristics according to the presence of total occlusion

	**No total occlusion (*n* = 8124)**	**Total occlusion (*n* = 1879)**	***P***
Sex (Women)	1956 (24.08)	341 (18.15)	< 0.001
Age, years	59 [51, 65]	57 [49, 64]	< 0.001
≥ 65 years	2260 (27.82)	464 (24.69)	0.006
BMI, kg/m^2^	25.82 [23.83, 27.76]	25.95 [24.21, 28.13]	< 0.001
Obesity	1883 (23.18)	497 (26.45)	0.003
Admission presentation			< 0.001
Acute myocardial infarction	1173 (14.43)	660 (31.12)	
Unstable angina	3571 (43.96)	617 (32.84)	
Chronic coronary syndrome	3380 (41.61)	602 (32.04)	
Smoking history	4605 (56.68)	1215 (64.66)	< 0.001
Diabetes	3236 (39.83)	752 (40.02)	0.880
Oral antidiabetic agents	1362 (42.09)	325 (43.22)	0.572
Insulin	874 (27.01)	180 (23.94)	0.085
Hypertension	5671 (69.81)	1270 (67.59)	0.060
Dyslipidemia	6113 (75.25)	1432 (76.21)	0.381
Peripheral artery disease	218 (2.68)	43 (2.29)	0.333
Chronic obstructive pulmonary disease	190 (2.34)	42 (2.24)	0.788
Prior myocardial infarction	1416 (17.43)	452 (24.06)	< 0.001
Prior stroke	869 (10.70)	211 (11.23)	0.503
Prior PCI	1827 (22.49)	471 (25.07)	0.017
eGFR, ml/min/1.73m^2^	118.44 [103.35, 133.66]	117.55 [101.24, 134.38]	0.082
LVEF, %	64 [60, 68]	61 [56, 65]	< 0.001
HbA1c, %	6.20 [5.80, 6.90]	6.20 [5.90, 7.00]	0.033
Fasting glucose, mmol/L	5.46 [5.92, 6.54]	5.65 [5.04, 7.11]	< 0.001
TC, mmol/L	4.05 [3.45, 4.79]	4.08 [3.42, 4.88]	0.358
TG, mmol/L	1.52 [1.13, 2.09]	1.57 [1.16, 2.15]	0.014
LDL-c, mmol/L	2.34 [1.86, 2.99]	2.39 [1.89, 3.08]	0.012
Non-HDL-c, mmol/L	3.00 [2.42, 3.74]	3.08 [2.47, 3.84]	< 0.001
HDL-c, mmol/L	1.00 [0.85, 1.19]	0.95 [0.81, 1.10]	< 0.001
Lp(a), mg/dL	17.83 [7.59, 40.21]	20.20 [8.63, 43.28]	< 0.001
ApoB, g/L	0.80 [0.66, 0.96]	0.81 [0.67, 1.00]	< 0.001
ApoA-1, g/L	1.32 [1.19, 1.50]	1.26 [1.14, 1.42]	< 0.001
Non-HDL-c/HDL-c	2.99 [2.29, 3.94]	3.27 [2.50, 4.27]	< 0.001
ApoB/apoA-1	0.60 [0.49, 0.74]	0.65 [0.52, 0.80]	< 0.001
Lesion location			
Left main coronary artery	196 (2.41)	44 (2.34)	0.856
Left anterior descending artery	7444 (91.63)	1617 (86.06)	< 0.001
Left circumflex artery	1268 (15.61)	446 (23.74)	< 0.001
Right coronary artery	1309 (16.11)	494 (26.29)	< 0.001

Values are presented as the median [interquartile range] or number (%)

*BMI* body mass index, *PCI* percutaneous coronary intervention, *eGFR* estimated glomerular filtration rate, *LVEF* left ventricular ejection fraction, *HbA1c* glycated hemoglobin

### Associations of lipid measures with total occlusion

For Lp(a), non-HDL-c/HDL-c, and apoB/apoA-1, the risks of total occlusion compared with the first quintiles were significantly higher through the subsequent quintiles (all *p* for trend < 0.001). For LDL-c, non-HDL-c, and apoB, the risks of total occlusion compared with the first quintiles were significantly higher only in the fifth quintiles. The risks of total occlusion in higher quintiles of TC and TG were not significantly different from the first quintiles. The three logistic regression models yielded consistent trends (Table 3).

**Table 3:** Associations of different lipid measures as continuous variables and in quintiles with total occlusion

	**Number (%)**	**Model 1**		**Model 2**		**Model 3**	
		**OR (95%CI)**	***P***	**OR (95%CI)**	***P***	**OR (95%CI)**	***P***
**TC, mmol/L**							
Per SD increase	—	1.050 (1.003, 1.099)	0.036	1.065 (1.017, 1.116)	0.007	1.072 (1.023, 1.123)	0.004
Q1: < 3.31	391 (19.54)	1.0 (Ref.)	—	1.0 (Ref.)	—	1.0 (Ref.)	—
Q2: 3.31 ≤ TC < 3.81	343 (17.19)	0.855 (0.728, 1.004)	0.056	0.868 (0.739, 1.019)	0.084	0.884 (0.752, 1.039)	0.136
Q3: 3.81 ≤ TC < 4.32	373 (18.76)	0.951 (0.812, 1.114)	0.533	0.980 (0.836, 1.148)	0.803	0.980 (0.835, 1.150)	0.803
Q4: 4.32 ≤ TC < 5.03	370 (18.25)	0.919 (0.785, 1.077)	0.297	0.958 (0.817, 1.123)	0.593	0.975 (0.830, 1.145)	0.756
Q5: ≥ 5.03	402 (20.18)	1.041 (0.891, 1.216)	0.612	1.090 (0.931, 1.276)	0.285	1.111 (0.948, 1.303)	0.195
p for trend	—	0.401	—	0.144	—	0.097	—
**TG, mmol/L**							
Per SD increase	—	1.043 (0.998, 1.089)	0.060	1.028 (0.983, 1.075)	0.223	1.016 (0.971, 1.065)	0.488
Q1: < 1.06	361 (17.95)	1.0 (Ref.)	—	1.0 (Ref.)	—	1.0 (Ref.)	—
Q2: 1.06 ≤ TG < 1.37	338 (17.15)	0.946 (0.803, 1.114)	0.506	0.938 (0.797, 1.106)	0.447	0.908 (0.769, 1.071)	0.251
Q3: 1.37 ≤ TG < 1.71	388 (19.23)	1.088 (0.928, 1.275)	0.298	1.080 (0.920, 1.267)	0.384	1.024 (0.871, 1.205)	0.770
Q4: 1.71 ≤ TG < 2.28	382 (19.01)	1.073 (0.915, 1.258)	0.385	1.053 (0.896, 1.237)	0.531	0.997 (0.847, 1.174)	0.973
Q5: ≥ 2.28	410 (20.56)	1.183 (1.011, 1.385)	0.036	1.138 (0.969, 1.336)	0.116	1.068 (0.906, 1.258)	0.436
p for trend	—	0.011	—	0.044	—	0.234	—
**LDL-c, mmol/L**							
Per SD increase	—	1.104 (1.047, 1.165)	< 0.001	1.114 (1.056, 1.176)	< 0.001	1.119 (1.060, 1.182)	< 0.001
Q1: < 1.76	355 (17.84)	1.0 (Ref.)	—	1.0 (Ref.)	—	1.0 (Ref.)	—
Q2: 1.76 ≤ LDL-c < 2.16	375 (18.66)	1.056 (0.900, 1.240)	0.503	1.062 (0.904, 1.247)	0.463	1.065 (0.905, 1.252)	0.448
Q3: 2.16 ≤ LDL-c < 2.59	360 (18.12)	1.019 (0.867, 1.198)	0.819	1.036 (0.881, 1.218)	0.671	1.045 (0.887, 1.231)	0.599
Q4: 2.59 ≤ LDL-c < 3.19	377 (18.64)	1.055 (0.899, 1.238)	0.514	1.077 (0.917, 1.265)	0.367	1.084 (0.921, 1.276)	0.331
Q5: ≥ 3.19	412 (20.67)	1.200 (1.025, 1.405)	0.023	1.232 (1.051, 1.444)	0.010	1.243 (1.059, 1.460)	0.008
p for trend	—	0.042	—	0.017	—	0.013	—
**Non-HDL-c, mmol/L**							
Per SD increase	—	1.110 (1.059, 1.163)	< 0.001	1.113 (1.061, 1.167)	< 0.001	1.113 (1.061, 1.168)	< 0.001
Q1: < 2.30	353 (17.50)	1.0 (Ref.)	—	1.0 (Ref.)	—	1.0 (Ref.)	—
Q2: 2.30 ≤ nHDL-c < 2.78	375 (19.02)	1.107 (0.943, 1.300)	0.216	1.106 (0.942, 1.300)	0.220	1.090 (0.926, 1.282)	0.300
Q3: 2.78 ≤ nHDL-c < 3.28	347 (17.09)	0.972 (0.826, 1.144)	0.732	0.982 (0.834, 1.157)	0.831	0.965 (0.818, 1.139)	0.674
Q4: 3.28 ≤ nHDL-c < 3.96	381 (19.24)	1.123 (0.957, 1.318)	0.155	1.132 (0.963, 1.330)	0.132	1.120 (0.952, 1.319)	0.172
Q5: ≥ 3.96	423 (21.11)	1.261 (1.078, 1.476)	0.004	1.275 (1.088, 1.494)	0.003	1.263 (1.076, 1.484)	0.004
p for trend	—	0.007	—	0.005	—	0.006	—
**Lp(a), mg/dL**							
Per SD increase	—	1.003 (1.001, 1.005)	0.002	1.003 (1.002, 1.005)	< 0.001	1.003 (1.002, 1.005)	< 0.001
Q1: < 6.27	323 (16.14)	1.0 (Ref.)	—	1.0 (Ref.)	—	1.0 (Ref.)	—
Q2: 6.27 ≤ lp(a) < 13.08	363 (18.15)	1.152 (0.977, 1.358)	0.092	1.177 (0.998, 1.389)	0.052	1.168 (0.989, 1.379)	0.068
Q3: 13.08 ≤ lp(a) < 25.21	387 (19.34)	1.246 (1.059, 1.466)	0.008	1.270 (1.079, 1.496)	0.004	1.263 (1.072, 1.489)	0.005
Q4: 25.21 ≤ lp(a) < 48.42	400 (20.00)	1.299 (1.105, 1.527)	0.002	1.331 (1.131, 1.565)	0.001	1.340 (1.138, 1.579)	0.001
Q5: ≥ 48.42	406 (20.29)	1.322 (1.125, 1.554)	0.001	1.391 (1.183, 1.637)	< 0.001	1.384 (1.175, 1.631)	0.001
p for trend	—	< 0.001	—	< 0.001	—	< 0.001	—
**ApoB, g/L**							
Per SD increase	—	1.603 (1.318, 1.949)	< 0.001	1.612 (1.323, 1.964)	< 0.001	1.605 (1.313, 1.961)	< 0.001
Q1: < 0.63	340 (17.03)	1.0 (Ref.)	—	1.0 (Ref.)	—	1.0 (Ref.)	—
Q2: 0.63 ≤ apoB < 0.75	369 (18.21)	1.085 (0.923, 1.277)	0.323	1.075 (0.914, 1.265)	0.384	1.078 (0.915, 1.270)	0.369
Q3: 0.75 ≤ apoB < 0.86	336 (17.68)	1.047 (0.887, 1.236)	0.587	1.041 (0.881, 1.230)	0.636	1.039 (0.878, 1.229)	0.658
Q4: 0.86 ≤ apoB < 1.02	391 (18.93)	1.138 (0.970, 1.336)	0.113	1.140 (0.970, 1.340)	0.111	1.126 (0.956, 1.325)	0.155
Q5: ≥ 1.02	443 (21.99)	1.373 (1.174, 1.607)	< 0.001	1.375 (1.173, 1.611)	< 0.001	1.373 (1.170, 1.613)	< 0.001
p for trend	—	< 0.001	—	< 0.001	—	< 0.001	—
**Non-HDL-c/HDL-c**							
Per SD increase	—	1.168 (1.129, 1.208)	< 0.001	1.149 (1.110, 1.190)	< 0.001	1.135 (1.095, 1.176)	< 0.001
Q1: < 2.17	288 (14.38)	1.0 (Ref.)	—	1.0 (Ref.)	—	1.0 (Ref.)	—
Q2: 2.17 ≤ ratio < 2.74	328 (16.37)	1.165 (0.981, 1.384)	0.081	1.136 (0.956, 1.350)	0.148	1.107 (0.930, 1.318)	0.252
Q3: 2.74 ≤ ratio < 3.39	398 (19.92)	1.481 (1.254, 1.749)	< 0.001	1.429 (1.209, 1.689)	< 0.001	1.380 (1.165, 1.635)	< 0.001
Q4: 3.39 ≤ ratio < 4.27	391 (19.57)	1.449 (1.226, 1.712)	< 0.001	1.390 (1.175, 1.644)	< 0.001	1.338 (1.129, 1.587)	< 0.001
Q5: ≥ 4.27	474 (23.70)	1.850 (1.573, 2.175)	< 0.001	1.727 (1.465, 2.037)	< 0.001	1.619 (1.368, 1.916)	< 0.001
p for trend	—	< 0.001	—	< 0.001	—	< 0.001	—
**ApoB/apoA-1**							
Per SD increase	—	3.030 (2.419, 3.795)	< 0.001	2.762 (2.196, 3.475)	< 0.001	2.590 (2.049, 3.274)	< 0.001
Q1: < 0.47	288 (14.31)	1.0 (Ref.)	—	1.0 (Ref.)	—	1.0 (Ref.)	—
Q2: 0.47 ≤ ratio < 0.57	326 (16.48)	1.181 (0.994, 1.403)	0.058	1.145 (0.963, 1.361)	0.125	1.126 (0.946, 1.341)	0.181
Q3: 0.57 ≤ ratio < 0.66	356 (17.73)	1.290 (1.089, 1.528)	0.003	1.243 (1.049, 1.474)	0.012	1.209 (1.018, 1.435)	0.030
Q4: 0.66 ≤ ratio < 0.79	410 (20.43)	1.537 (1.303, 1.813)	< 0.001	1.468 (1.243, 1.734)	< 0.001	1.427 (1.206, 1.688)	< 0.001
Q5: ≥ 0.79	499 (24.97)	1.993 (1.697, 2.340)	< 0.001	1.871 (1.590, 2.202)	< 0.001	1.787 (1.514, 2.109)	< 0.001
p for trend	—	< 0.001	—	< 0.001	—	< 0.001	—

Model 1 was the crude model. Model 2 was adjusted for sex and age. Model 3 was adjusted for sex, age, body mass index, hypertension, diabetes, prior myocardial infarction, prior percutaneous coronary intervention, smoking history, and admission presentation

*OR* odds ratio, *CI* confidence interval, *SD* standard deviation, *Q* quintile, *Ref* reference

**Fig. 1:**
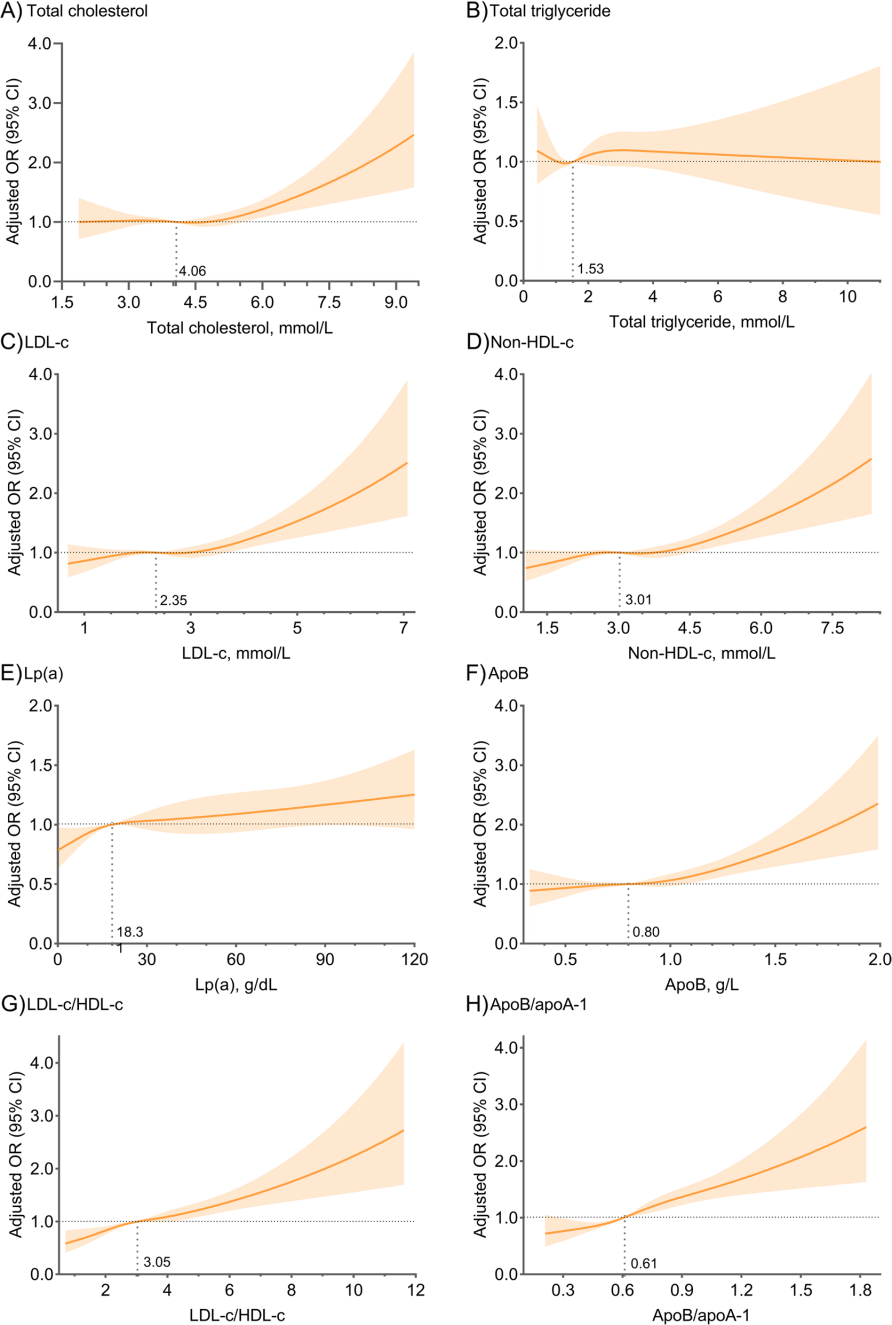
Adjusted restricted cubic spline curves of different lipid measures. Adjusted for sex, age, body mass index, hypertension, diabetes, prior myocardial infarction, prior percutaneous coronary intervention, smoking history, and admission presentation. OR, odds ratio; CI, confidence interval; LDL-c, low-density lipoprotein cholesterol; HDL-c, high-density lipoprotein cholesterol; Lp(a), lipoprotein (a); apo, apolipoprotein

**Fig. 2:**
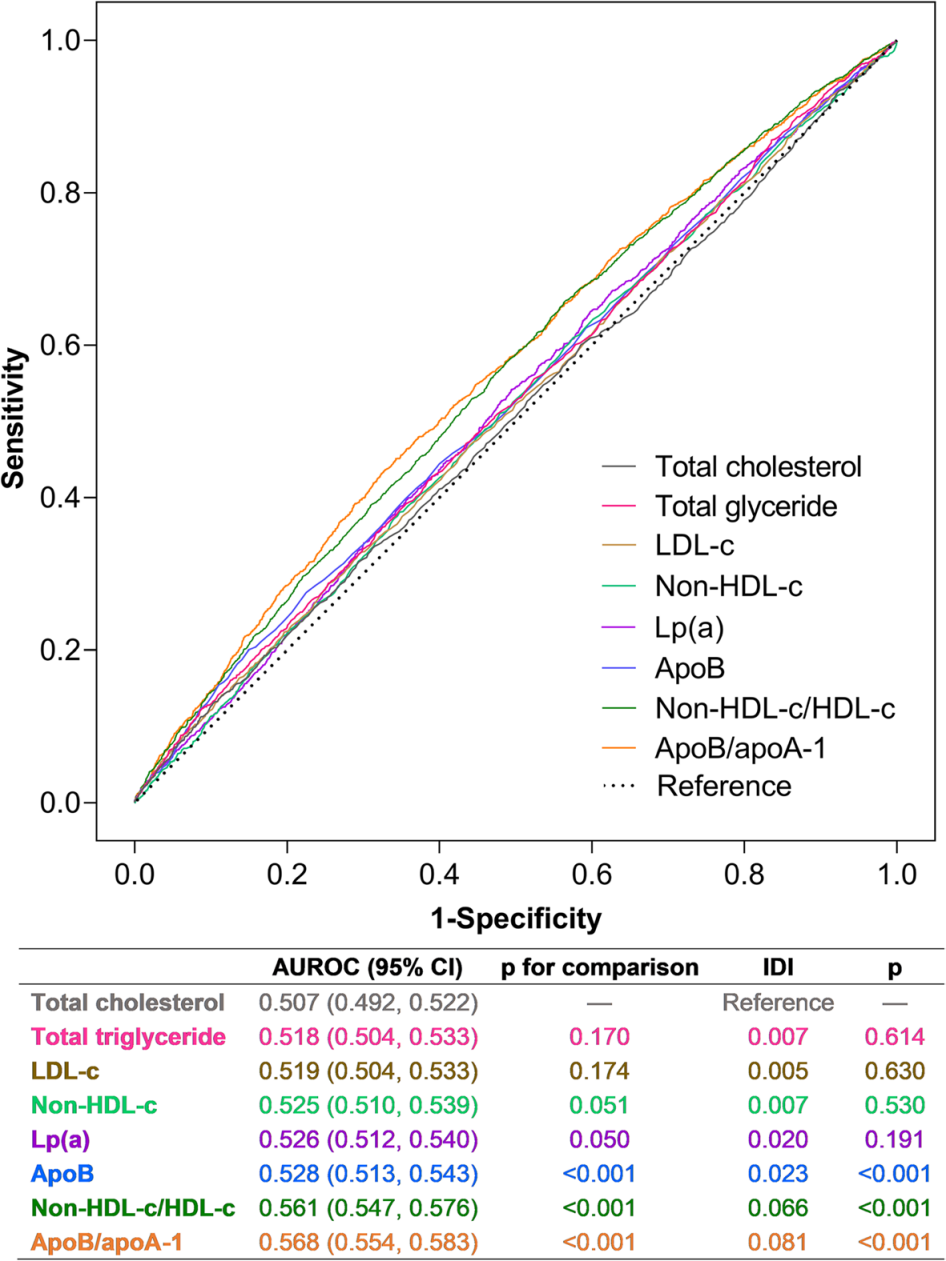
ROC curves of different lipid measures for prediction of total occlusion. ROC, receiver operating characteristic curve; apo, apolipoprotein; LDL-c, low-density lipoprotein cholesterol; HDL-c, high-density lipoprotein cholesterol; lp(a), lipoprotein (a); AUROC, area under the receiver operating characteristic curve; IDI, integrated discrimination index; CI, confidence interval

Restricted cubic spline curves indicate significantly positive linear relations between lipid measure levels and the risk of total occlusion except for TG. Notably, the 95% CIs for the ORs included the null value when either the Lp(a) level was greater than the median or the levels of other single lipid measures were less than the medians (Fig. 1, Table 3). The AUROCs of apoB/apoA-1 and non-HDL-c/HDL-c were significantly greater than those of other measures, with integrated discrimination improvements of 8.1% and 6.6%, respectively (*P* < 0.001) (Fig. 2).

Only ApoB/apoA-1 and non-HDL-c/HDL-c were robustly associated with total occlusion in all subgroups. ROC illustrated similar results that the two lipid ratios confer better predictive power of total occlusion than other single lipid measures in all subgroups (see Supplemental Tables 9–10 in Additional File). Sensitivity analysis including only patients with acute coronary disease yielded consistent results with the main analysis (see Supplemental Tables 11–12 in Additional File).

### Discordance analysis of apoB/apoA-1 and non-HDL-c/HDL-c

The above results demonstrated that apoB/apoA-1 and non-HDL-c/HDL-c were better predictors of total occlusion. However, according to our definition, the two ratios were discordant in 3253 (32.52%) patients in the study population.

**Fig. 3:**
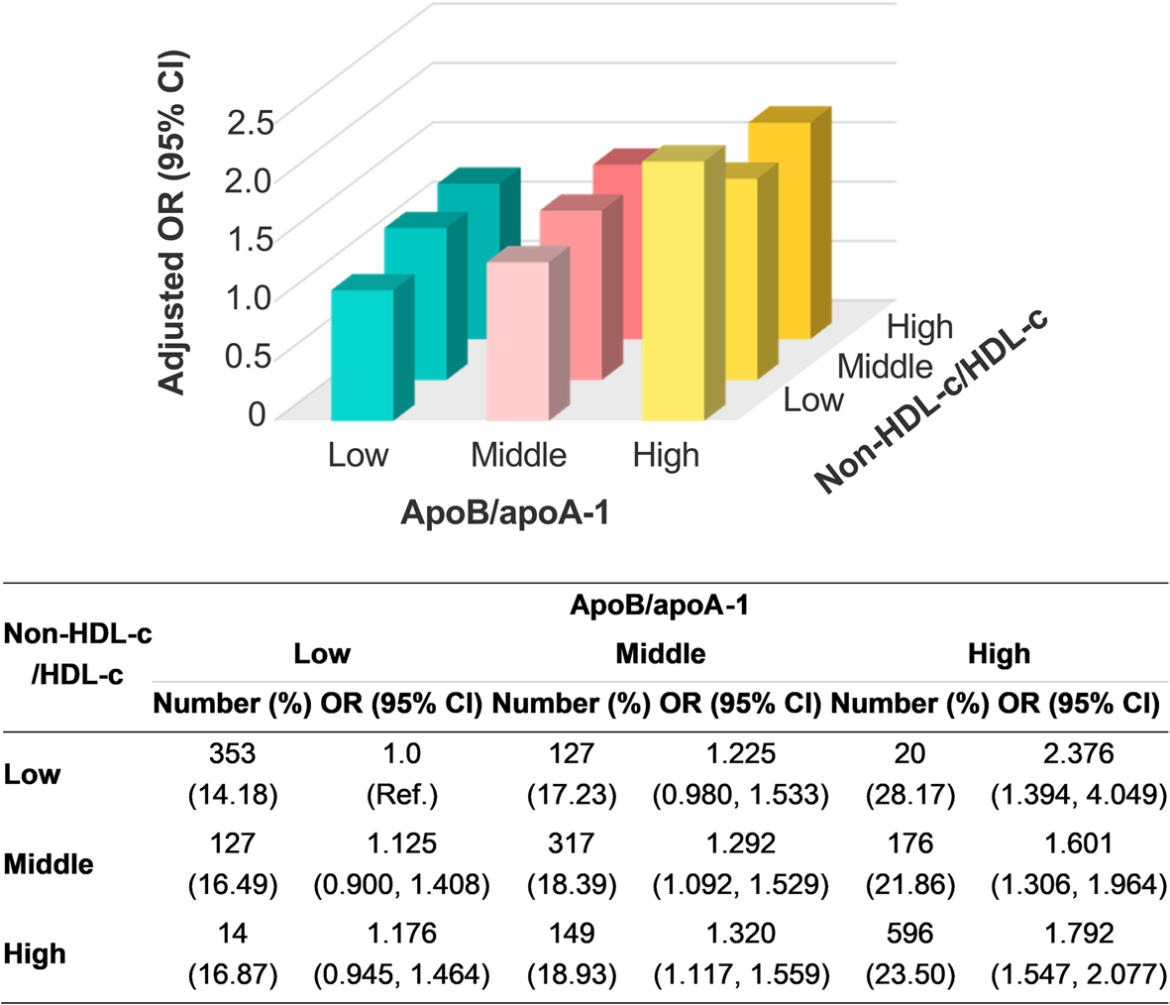
Incidence and adjusted OR of total occlusion by concordance/discordance groups between apoB/apoA-1 and non-HDL-c/HDL-c**.** Adjusted for sex, age, body mass index, hypertension, diabetes, prior myocardial infarction, prior percutaneous coronary intervention, smoking history, and admission presentation. OR, odds ratio; apo, apolipoprotein; LDL-c, low-density lipoprotein cholesterol; HDL-c, high-density lipoprotein cholesterol; CI, confidence interval; Ref., reference

Elevation in the apoB/apoA-1 tertile significantly increased the incidence and the risk of total occlusion at a given non-HDL-c/HDL-c tertile. A trend that elevation in the non-HDL-c/HDL-c tertile slightly increased the incidence and the risk of total occlusion was observed at the low or middle level of apoB/apoA-1. However, the trend disappeared at the high apoB-apoA-1 level. Patients in the high-apoB/apoA-1-low-non-HDL-c/HDL-c group had the highest incidence and risk of total occlusion (Fig. 3). Models 1 and 2 obtained consistent results (data not shown). Consistent trends were observed in the sensitivity analysis (see Supplemental Table 13 in Additional file).

## Discussion

In this study, we investigated the association of lipid profiles with total occlusion in established CAD patients to seek new possibilities for the early diagnosis of total occlusion. We observed that total occlusion was frequent in the study population; patients with total occlusion had significantly higher levels of lipids and lipid ratios; lipid ratios were more stable predictors of total occlusion than single lipid measures; in particular, apoB/apoA-1 was more sensitive than non-HDL-c/HDL-c and should be highlighted in clinical practice.

### Single lipid measures and total occlusion

ApoB, LDL-c, and non-HDL-c are strongly correlated with each other. The plasma apoB level is approximately equal to the sum of triglyceride-rich very-low-density lipoprotein, cholesterol-rich LDL, and Lp(a), representing the number of circulating atherogenic particles [12]. We observed that the OR of apoB for total occlusion was higher than that of LDL-c and non-HDL-c. Previous Mendelian randomization studies and discordance analyses have further determined that apoB is more accurate as a marker of coronary calcification and cardiovascular disease than LDL-c or non-HDL-c [12–14]. These findings confirm the widely accepted view that the number of atherogenic particles is more relative to atherosclerosis than the concentration of circulating cholesterol, as only apoB-containing particles < 70 nm in diameter can enter and be retained in the arterial wall, thereby initiating and driving atherosclerosis.

Lp(a) was also observed to be associated with the risk of total occlusion. A possible mechanism is that Lp(a) is related to the progression of low-attenuation plaque (a quantitative marker of necrotic core) on coronary computed tomography angiography in established CAD patients [15]. Low attenuation is an independent predictor of plaque progression to chronic total occlusion [16], and a necrotic core is related to the increased propensity of plaque rupture and subsequent acute total occlusion [15]. However, the OR per 1 SD increase in Lp(a) was only 1.003 (95% CI: 1.002–1.005). This phenomenon could be explained by the fact that the effect of Lp(a) on CAD risk is proportional to the absolute change in plasma Lp(a) levels [17, 18], while a 1-SD change is too small to provide a clinically meaningful reduction in the risk of total occlusion.

Sensitivity analysis in ACS patients generated consistent results with the findings from the overall CAD patients, indicating that transient fluctuation of lipid levels under acute conditions may not change the association of lipid profile with total occlusion. In addition, a study reported high-sensitivity cardiac troponin T as a predictor of acute total occlusion [5]. However, we believe that cardiac troponin, an acute-phase reactant of myocardial injury, is a consequence rather than a cause of acute occlusion.

### Lipid ratios and total occlusion

Compared with single lipid measures, apoB/apoA-1 and non-HDL-c/HDL-c had higher and significant ORs, and the superiority of the two ratios was robust in all subgroups. Conversely, single lipid measures lost predictive power in some quintiles or subgroups, suggesting that lipid ratios are more stable indicators of total occlusion. Previous studies support that lipid ratios are superior to single lipid measures in predicting vulnerable plaque and MI [7, 8, 19]. HDL can reverse cholesterol transport and is inversely correlated with cardiovascular risk. An elevation in HDL is associated with obstructive coronary lesions for a given LDL level. ApoA-1 is the major component of HDL. Reduction in apoA-1 potentiates the impact of apoB on major cardiovascular events at any apoB level [20, 21]. Accordingly, lipid ratios reflect the balance between risk and protective factors for atherosclerosis, providing more comprehensive information on atherosclerosis risk than single lipid measures.

For the comparison of apoB/apoA-1 and non-HDL-c/HDL-c, the risk of total occlusion per 1-SD increase in apoB/apoA-1 was over twice as high as non-HDL-c/HDL-c, suggesting that a 1-SD decrease in apoB/apoA-1 can produce a greater benefit than non-HDL-c/HDL-c. The finding was valid in all subgroups. Previous studies have yielded similar results that apoB/apoA-1 is better than other lipid ratios in predicting coronary severity and MI [22–25]. The AUROCs illustrated comparable prediction performance of ApoB/apoA-1 and non-HDL-c/HDL-c. Thus, we used discordance analysis to discriminate between the two strongly correlated ratios. We observed that elevation in the apoB/apoA-1 tertile significantly increased the incidence and the risk of total occlusion at a given non-HDL-c/HDL-c tertile but not vice versa, demonstrating that even at a low non-HDL-c/HDL-c level, a further reduction in apoB/apoA-1 can still lessen the risk of total occlusion. Two possible explanations are as follows. First, only apoB-containing particles can cross the arterial wall; when plasma cholesterol is constant, a higher apoB level means more circulating cholesterol can be transferred, trapped, and deposed. Thus, elevation in apoB aggravates the risk of total occlusion regardless of non-HDL-c level. However, once within the arterial wall, smaller apoB particles with less cholesterol are more hazardous because they have a greater tendency to be trapped than larger cholesterol-rich apoB particles [12, 23], which proves our finding that patients in the high-apoB/apoA-1-low-non-HDL-c/HDL-c group experienced the highest risk of total occlusion. Second, in addition to reversing cholesterol transport, apoA-1 has antioxidation and anti-inflammation abilities and is associated with insulin resistance. Elevation in apoA-1 may bring more protective effects than HDL [26]. Moreover, apoB and apoA-1 are directly assayed and hardly affected by fasting status, making apoB/apoA-1 a superior indicator to non-HDL-c/HDL-c in clinical practice.

Altogether, the lipid profile drives the development of CAD, a continuous and evolving process from lipid deposition and plaque growth to ischemic events. Our work and studies applying coronary severity as the endpoint are mutually supportive. In conclusion, we explored the association of lipid profiles with total occlusion and discovered the advantages of lipid ratios, especially apoB/apoA-1, in predicting total occlusion. Our findings suggest that apoB/apoA-1 should be monitored routinely to identify patients at high risk of total occlusion and to guide lipid-lowering treatment for CAD patients to delay disease progression. In addition, approximately a quarter of non-ST-segment elevation myocardial infarction is caused by a totally occluded culprit vessel [1]. These patients have adverse outcomes and need immediate revascularization. However, the early noninvasive identification of these high-risk patients is unsatisfactory. ApoB/apoA-1 may be a potential indicator for identifying total occlusion of a culprit vessel in non-ST-segment elevation myocardial infarction patients at admission. Furthermore, a study preliminarily proposed three risk levels of apoB/apoA-1: low risk, 0.2–0.6; intermediate risk, 0.61–0.9; and high risk, 0.91–5.0 [21]. A certain cutoff value should be established in future research.

### Study strengths and limitations

The study provides evidence on the association between lipid profile and total occlusion in established CAD patients, adding novel insights to the prediction of total occlusion. Other strengths include the large population and low missingness rate (< 2.80%). The study limitations should be noted. First, although we made great efforts to minimize potential confounders, the observational design of this study raises concerns about residual confounding by some unknown and unmeasured factors associated with total occlusion. Second, the single-center nature of this study restricts its generalizability. Third, the lipid profile was assayed only once before the procedure. Repeated measurements estimating long-term average lipid concentrations were unavailable. Thus, we cannot rule out the influence of acute stress on the lipid profile, as lipid levels tend to decrease under acute conditions. Fourth, data on the preadmission use of lipid-modifying drugs were not collected, so we could not adjust for the plaque stabilization effect of lipid-lowering drugs such as statins. Last, we could not distinguish between acute or chronic total occlusion. Nevertheless, our findings are reliable because the precursors of chronic total occlusion and AMI may be more similar than their clinical manifestations [27].

## Conclusions

ApoB/apoA-1 confers better predictive power of total occlusion than non-HDL-c/HDL-c and single lipid measures in established CAD patients. Routine apoB/apoA-1 monitoring for CAD patients can help identify individuals at high-risk of total occlusion and guide lipid-lowering therapy to delay disease progression.

## Supplementary Information

**Additional file 1: Supplemental Methods. Supplemental Table1.** Baseline characteristics according to quintiles of total cholesterol. **Supplemental Table2.** Baseline characteristics according to quintiles of total triglyceride. **Supplemental Table3.** Baseline characteristics according to quintiles of LDL-c. **Supplemental Table4.** Baseline characteristics according to quintiles of non-HDL-c. **Supplemental Table5.** Baseline characteristics according to quintiles of lp(a). **Supplemental Table6.** Baseline characteristics according to quintiles of apoB. **Supplemental Table7.** Baseline characteristics according to quintiles of non-HDL-c/HDL-c. **Supplemental Table8.** Baseline characteristics according to quintiles of apoB/apoA-1. **Supplemental Table9.** Associations of different lipid measures as continuous variables with total occlusion by subgroups. **Supplemental Table10**. AUROC of each lipid measure by subgroups. **Supplemental Table11.** Associations of different lipid measures with total occlusion in patients with acute coronary syndrome. **Supplemental Table12**. Predictive value of different lipid measures for total occlusion in patients with acute coronary syndrome. **Supplemental Table13**. Discordance analysis between apoB/apoA-1 and non-HDL-c/HDL-c in patients with acute coronary syndrome. **Supplemental Figure1.** Distribution of each lipid measure.

## Data Availability

The data that support the findings of this study are available from the Information Center of Fuwai Hospital, but restrictions apply to the availability of these data, which were used under license for the current study and are not publicly available. Data are, however, available from the authors upon reasonable request and with permission of the Information Center of Fuwai Hospital.

## Abbreviations

CAD: Coronary artery disease
TC: Total cholesterol
TG: Total triglyceride
LDL-c: Low-density lipoprotein cholesterol
HDL-c: High-density lipoprotein cholesterol
Lp(a): Lipoprotein (a)
apo: Apolipoprotein
AUROC: Area under the receiver operating characteristic curve
AMI: Acute myocardial infarction
PCI: Percutaneous coronary intervention
ROC: Receiver operating characteristic curve
OR: Odds ratio
CI: Confidence interval
SD: Standard deviation
