# Supplementary material for: Associations of lipid measures with total occlusion in patients with established coronary artery disease: a cross-sectional study
Source: Lipids Health Dis. 2022 Nov 11;21:118. doi: 10.1186/s12944-022-01733-8 (PMC9650853; doi:10.1186/s12944-022-01733-8)
Supplement: Supplementary file 1 — Additional file 1: Supplemental Methods. Supplemental Table1. Baseline characteristics according to quintiles of total cholesterol. Supplemental Table2. Baseline characteristics according to quintiles of total triglyceride. Supplemental Table3. Baseline characteristics according to quintiles of LDL-c. Supplemental Table4. Baseline characteristics according to quintiles of non-HDL-c. Supplemental Table5. Baseline characteristics according to quintiles of lp(a). Supplemental Table6. Baseline characteristics according to quintiles of apoB. Supplemental Table7. Baseline characteristics according to quintiles of non-HDL-c/HDL-c. Supplemental Table8. Baseline characteristics according to quintiles of apoB/apoA-1. Supplemental Table9. Associations of different lipid measures as continuous variables with total occlusion by subgroups. Supplemental Table10. AUROC of each lipid measure by subgroups. Supplemental Table11. Associations of different lipid measures with total occlusion in patients with acute coronary syndrome. Supplemental Table12. Predictive value of different lipid measures for total occlusion in patients with acute coronary syndrome. Supplemental Table13. Discordance analysis between apoB/apoA-1 and non-HDL-c/HDL-c in patients with acute coronary syndrome. Supplemental Figure1. Distribution of each lipid measure. [file 12944_2022_1733_MOESM1_ESM.docx]

**Additional file for**

**Associations of lipid measures with total occlusion in patients with established coronary artery disease: a cross-sectional study**

[Supplemental Methods 2](#_Toc114857156)

[Supplemental Table1. Baseline characteristics according to quintiles of total cholesterol. 3](#_Toc114857157)

[Supplemental Table2. Baseline characteristics according to quintiles of total triglyceride. 5](#_Toc114857158)

[Supplemental Table3. Baseline characteristics according to quintiles of LDL-c. 7](#_Toc114857159)

[Supplemental Table4. Baseline characteristics according to quintiles of non-HDL-c. 9](#_Toc114857160)

[Supplemental Table5. Baseline characteristics according to quintiles of lp(a). 11](#_Toc114857161)

[Supplemental Table6. Baseline characteristics according to quintiles of apoB. 13](#_Toc114857162)

[Supplemental Table7. Baseline characteristics according to quintiles of non-HDL-c/HDL-c. 15](#_Toc114857163)

[Supplemental Table8. Baseline characteristics according to quintiles of apoB/apoA-1. 17](#_Toc114857164)

[Supplemental Table9. Associations of different lipid measures as continuous variables with total occlusion by subgroups. 19](#_Toc114857165)

[Supplemental Table10. AUROC of each lipid measure by subgroups. 20](#_Toc114857166)

[Supplemental Table11. Associations of different lipid measures with total occlusion in patients with acute coronary syndrome. 21](#_Toc114857167)

[Supplemental Table12. Predictive value of different lipid measures for total occlusion in patients with acute coronary syndrome. 23](#_Toc114857168)

[Supplemental Table13. Discordance analysis between apoB/apoA-1 and non-HDL-c/HDL-c in patients with acute coronary syndrome. 24](#_Toc114857169)

[Supplemental Figure1. Distribution of each lipid measure. 25](#_Toc114857170)

[Supplemental Reference 26](#_Toc114857171)

# Supplemental Methods

The study sample size was calculated by easyROC web-tool. [1] [2] The study sought to examine the association between lipid profile and total occlusion in established patients with coronary artery disease and compare the prediction for total occlusion of different lipid measures. The prevalence of total occlusion in our study population was previously determined (19%), thus, the allocation ratio was calculated as (1-proportion of patients with total occlusion)/proportion of patients with total occlusion. The Type I error was set as 0.05 and the Type II error was set as 0.01. The null hypothesis was an AUC of 0.50. According to our previous investigation, an AUC of 0.55 was set as reference. An AUC of 0.60 was considered to be significantly different from the reference. The estimated sample size was 8032 patients.

# Supplemental Table1. Baseline characteristics according to quintiles of total cholesterol.

|  | **Total cholesterol, mmol/L** | | | | | **P for trend** |
| --- | --- | --- | --- | --- | --- | --- |
|  | **Q1 (n=2001)**  **<3.31** | **Q2 (n=1995)**  **3.31≤TC<3.81** | **Q3 (n=1988)**  **3.81≤TC<4.32** | **Q4 (n=2027)**  **4.32≤TC<5.03** | **Q5 (n=1992)**  **≥5.03** |  |
| Sex (Women) | 272 (13.59) | 368 (18.45) | 471 (23.69) | 555 (27.38) | 631 (31.68) | <0.001 |
| Age, years | 58 [50, 65] | 59 [50, 66] | 59 [51, 65] | 58 [51, 66] | 58 [50, 64] | 0.078 |
| ≥65 years | 552 (27.59) | 573 (28.72) | 545 (27.41) | 563 (27.78) | 491 (24.65) | 0.031 |
| BMI, kg/m^2^ | 25.89 [24.01, 27.76] | 25.71 [23.66, 27.68] | 25.93 [23.88, 27.77] | 25.82 [23.76, 27.76] | 25.97 [24.09, 28.10] | 0.006 |
| Obesity | 461 (23.04) | 441 (22.11) | 479 (24.10) | 468 (23.09) | 531 (26.66) | 0.009 |
| Admission presentation |  |  |  |  |  |  |
| AMI | 325 (16.24) | 355 (17.79) | 399 (20.07) | 420 (20.72) | 334 (16.77) | <0.001 |
| Unstable angina | 818 (40.88) | 818 (41.00) | 852 (42.86) | 838 (41.34) | 862 (43.27) | <0.001 |
| CCS | 858 (42.88) | 822 (41.20) | 737 (37.07) | 769 (37.94) | 796 (39.96) | <0.001 |
| Smoking history | 1237 (61.82) | 1212 (60.75) | 1161 (58.40) | 1157 (57.08) | 1053 (52.86) | <0.001 |
| Diabetes | 842 (42.08) | 808 (40.50) | 779 (39.19) | 739 (36.56) | 820 (41.17) | 0.087 |
| Oral antidiabetic agents | 424 (50.36) | 370 (45.79) | 326 (41.85) | 284 (38.43) | 283 (34.51) | <0.001 |
| Insulin | 229 (27.20) | 219 (27.10) | 200 (25.67) | 202 (27.33) | 204 (24.88) | 0.352 |
| Hypertension | 1375 (68.72) | 1398 (70.08) | 1397 (70.27) | 1396 (68.87) | 1375 (69.03) | 0.857 |
| Dyslipidemia | 1433 (71.61) | 1433 (71.83) | 1463 (73.59) | 1498 (73.90) | 1718 (86.25) | <0.001 |
| Peripheral artery disease | 58 (2.90) | 60 (3.01) | 52 (2.62) | 49 (2.42) | 42 (2.11) | 0.055 |
| COPD | 51 (2.55) | 46 (2.31) | 52 (2.52) | 43 (2.12) | 40 (2.01) | 0.234 |
| Prior myocardial infarction | 506 (25.29) | 396 (19.85) | 358 (10.01) | 300 (14.80) | 308 (15.46) | <0.001 |
| Prior stroke | 233 (11.64) | 221 (11.08) | 228 (11.47) | 215 (10.61) | 183 (9.19) | 0.014 |
| Prior PCI | 568 (28.39) | 495 (24.81) | 455 (22.89) | 402 (19.83) | 378 (18.98) | <0.001 |
| eGFR, ml/min/1.73m^2^ | 118.82 [103.06, 134.49] | 119.08 [104.34, 134.06] | 118.21 [101.99, 134.19] | 118.45 [102.95, 133.59] | 116.86 [101.80, 133.05] | 0.032 |
| LVEF, % | 64 [60, 67] | 64 [60, 67] | 64 [60, 67] | 64 [60, 67] | 64 [60, 67] | 0.677 |
| HbA1c, % | 6.20 [5.80, 6.90] | 6.20 [5.90, 6.90] | 6.20 [5.80, 6.90] | 6.20 [5.80, 6.90] | 6.20 [5.90, 7.10] | <0.001 |
| Fasting glucose, mmol/L | 5.31 [4.83, 6.39] | 5.40 [4.89, 6.49] | 5.53 [4.94, 6.67] | 5.53 [4.97, 6.63] | 5.69 [5.09, 7.07] | <0.001 |
| Total cholesterol, mmol/L | 2.99 [2.73, 3.16] | 3.57 [3.45, 3.69] | 4.05 [3.92, 4.19] | 4.62 [4.46, 4.81] | 5.65 [5.31, 6.16] | <0.001 |
| Total triglyceride, mmol/L | 1.21 [0.94, 1.57] | 1.39 [1.06, 1.82] | 1.53 [1.18, 2.07] | 1.73 [1.28, 2.30] | 1.97 [1.48, 2.80] | <0.001 |
| LDL-c, mmol/L | 1.554 [1.33,1.73] | 2.00 [1.83, 2.17] | 2.38 [2.19, 2.57] | 2.87 [2.63, 3.10] | 3.68 [3.35, 4.18] | <0.001 |
| non-HDL-c, mmol/L | 2.04 [1.82, 2.26] | 2.58 [2.40, 2.75] | 3.04 [2.83, 3.23] | 3.58 [3.35, 3.80] | 4.55 [4.20, 5.06] | <0.001 |
| HDL-c, mmol/L | 0.88 [0.75, 1.02] | 0.97 [0.83, 1.14] | 1.01 [0.85, 1.19] | 1.03 [0.88, 1.23] | 1.09 [0.93, 1.30] | <0.001 |
| Lp(a), mg/dL | 13.50 [6.06, 31.26] | 17.15 [7.39, 37.01] | 19.23 [8.44, 42.51] | 20.89 [8.51, 44.75] | 21.41 [9.10, 47.81] | <0.001 |
| ApoB, g/L | 0.58 [0.52, 0.65] | 0.70 [0.64, 0.77] | 0.79 [0.73, 0.87] | 0.92 [0.84, 1.00] | 1.13 [1.01, 1.29] | <0.001 |
| ApoA-1, g/L | 1.19 [1.08, 1.30] | 1.29 [1.17, 1.42] | 1.33 [1.20, 1.49] | 1.36 [1.22, 1.54] | 1.43 [1.28, 1.62] | <0.001 |
| Non-HDL-c/HDL-c | 2.31 [1.84, 2.82] | 2.65 [2.14, 3.29] | 3.01 [2.40, 3.80] | 3.49 [2.77, 4.29] | 4.29 [3.43, 5.29] | <0.001 |
| ApoB/apoA-1 | 0.49 [0.42, 0.58] | 0.55 [0.46, 0.64] | 0.60 [0.50, 0.72] | 0.68 [0.57, 0.80] | 0.80 [0.66, 0.96] | <0.001 |

Values are presented as median [interquartile range] or number (%).

Q, quintile; TC, total cholesterol; BMI, body mass index; AMI, acute myocardial infarction; CCS, chronic coronary syndrome; COPD, chronic obstructive pulmonary disease; PCI, percutaneous coronary intervention; eGFR, estimated glomerular filtration rate; LVEF, left ventricular ejection fraction; HbA1c, glycated hemoglobin; LDL-c, low-density lipoprotein cholesterol; HDL-c, high-density lipoprotein cholesterol; lp(a), lipoprotein (a); apo, apolipoprotein.

# Supplemental Table2. Baseline characteristics according to quintiles of total triglyceride.

|  | **Total triglyceride, mmol/L** | | | | | **P for trend** |
| --- | --- | --- | --- | --- | --- | --- |
|  | **Q1 (n=2011)**  **<1.06** | **Q2 (n=1971)**  **1.06≤TG<1.37** | **Q3 (n=2018)**  **1.37≤TG<1.71** | **Q4 (n=2009)**  **1.71≤TG<2.28** | **Q5 (n=1994)**  **≥2.28** |  |
| Sex (Women) | 403 (20.04) | 426 (21.61) | 504 (24.98) | 495 (24.64) | 469 (23.52) | 0.001 |
| Age, years | 61 [54, 69] | 60 [53, 67] | 58 [51, 65] | 58 [50, 64] | 55 [48, 62] | <0.001 |
| ≥65 years | 749 (37.25) | 610 (30.95) | 548 (27.16) | 472 (23.49) | 345 (17.30) | <0.001 |
| BMI, kg/m^2^ | 24.88 [22.84, 26.94] | 25.40 [23.59, 27.64] | 25.90 [23.88, 27.96] | 26.12 [24.22, 28.08] | 26.57 [24.62, 28.41] | <0.001 |
| Obesity | 333 (16.56) | 405 (20.55) | 503 (24.93) | 517 (25.73) | 622 (31.19) | <0.001 |
| Admission presentation |  |  |  |  |  |  |
| AMI | 316 (15.71) | 338 (17.15) | 376 (18.64) | 421 (20.96) | 382 (19.15) | <0.001 |
| Unstable angina | 830 (41.27) | 856 (43.43) | 847 (41.97) | 821 (40.87) | 834 (41.83) | <0.001 |
| CCS | 865 (43.01) | 777 (39.42) | 795 (39.40) | 767 (38.18) | 778 (39.02) | <0.001 |
| Smoking history | 1146 (56.99) | 1125 (57.08) | 1139 (56.44) | 1167 (58.09) | 1243 (62.34) | 0.001 |
| Diabetes | 697 (34.66) | 741 (37.60) | 821 (40.68) | 808 (40.22) | 921 (46.19) | <0.001 |
| Oral antidiabetic agents | 338 (48.49) | 329 (44.40) | 345 (42.02) | 306 (37.87) | 369 (40.07) | <0.001 |
| Insulin | 196 (28.12) | 199 (26.86) | 208 (25.34) | 214 (26.49) | 237 (25.73) | 0.320 |
| Hypertension | 1359 (67.58) | 1361 (69.05) | 1401 (69.43) | 1393 (69.34) | 1427 (71.57) | 0.011 |
| Dyslipidemia | 1248 (62.06) | 1334 (67.68) | 1453 (72.00) | 1522 (75.76) | 1988 (99.70) | <0.001 |
| Peripheral artery disease | 65 (3.23) | 41 (2.08) | 44 (2.18) | 48 (2.39) | 63 (3.16) | 0.898 |
| COPD | 50 (2.49) | 54 (2.74) | 49 (2.43) | 41 (2.04) | 38 (1.91) | 0.082 |
| Prior myocardial infarction | 390 (19.39) | 375 (19.03) | 400 (19.82) | 351 (17.47) | 352 (17.65) | 0.068 |
| Prior stroke | 232 (11.54) | 249 (12.63) | 215 (10.65) | 192 (9.56) | 192 (9.63) | 0.002 |
| Prior PCI | 503 (25.01) | 460 (23.34) | 461 (22.84) | 438 (21.80) | 436 (21.87) | 0.008 |
| eGFR, ml/min/1.73m^2^ | 119.16 [104.25, 134.41] | 118.12 [102.56, 133.80] | 118.23 [102.26, 134.59] | 117.78 [101.93, 131.68] | 118.43 [103.25, 134.32] | 0.025 |
| LVEF, % | 64 [60, 68] | 64 [60, 67] | 64 [60, 67] | 64 [60, 67] | 63 [60, 67] | 0.082 |
| HbA1c, % | 6.10 [5.80, 6.60] | 6.20 [5.80, 6.80] | 6.20 [5.90, 7.00] | 6.20 [5.80, 7.00] | 6.30 [5.90, 7.40] | <0.001 |
| Fasting glucose, mmol/L | 5.31 [4.84, 6.27] | 5.42 [4.90, 6.42] | 5.49 [4.94, 6.62] | 5.53 [4.97, 6.72] | 5.75 [5.07, 7.20] | <0.001 |
| Total cholesterol, mmol/L | 3.55 [3.04, 4.15] | 3.84 [3.30, 4.47] | 4.03 [3.48, 4.74] | 4.28 [3.66, 4.95] | 4.66 [4.06, 5.50] | <0.001 |
| Total triglyceride, mmol/L | 0.89 [0.77, 0.98] | 1.21 [1.14, 1.29] | 1.53 [1.44, 1.61] | 1.95 [1.83, 2.10] | 2.90 [2.52, 3.58] | <0.001 |
| LDL-c, mmol/L | 2.00 [1.63, 2.53] | 2.27 [1.82, 2.86] | 2.41 [1.95, 3.09] | 2.56 [2.02, 3.21] | 2.62 [2.05, 3.27] | <0.001 |
| non-HDL-c, mmol/L | 2.37 [1.98, 2.89] | 2.76 [2.30, 3.38] | 3.00 [2.52, 3.67] | 3.28 [2.72, 3.94] | 3.74 [3.17, 4.53] | <0.001 |
| HDL-c, mmol/L | 1.11 [0.94, 1.33] | 1.03 [0.88, 1.22] | 1.00 [0.85, 1.17] | 0.95 [0.81, 1.12] | 0.90 [0.77, 1.05] | <0.001 |
| Lp(a), mg/dL | 18.74 [8.51, 40.74] | 20.22 [9.09, 42.37] | 21.22 [8.96, 47.76] | 18.40 [8.13, 41.15] | 12.71 [5.44, 31.71] | <0.001 |
| ApoB, g/L | 0.66 [0.57, 0.78] | 0.75 [0.64, 0.89] | 0.80 [0.68, 0.97] | 0.87 [0.73, 1.04] | 0.95 [0.80, 1.12] | <0.001 |
| ApoA-1, g/L | 1.32 [1.18, 1.51] | 1.31 [1.17, 1.48] | 1.30 [1.17, 1.48] | 1.31 [1.17, 1.46] | 1.32 [1.20, 1.47] | 0.262 |
| Non-HDL-c/HDL-c | 2.17 [1.69, 2.72] | 2.68 [2.15, 3.37] | 3.05 [243, 3.84] | 3.44 [2.75, 4.26] | 4.20 [3.40, 5.13] | <0.001 |
| ApoB/apoA-1 | 0.50 [0.41, 0.60] | 0.57 [0.48, 0.69] | 0.61 [0.50, 0.74] | 0.66 [0.54, 0.80] | 0.72 [0.59, 0.86] | <0.001 |

Values are presented as median [interquartile range] or number (%).

Q, quintile; TG, total triglyceride; BMI, body mass index; AMI, acute myocardial infarction; CCS, chronic coronary syndrome; COPD, chronic obstructive pulmonary disease; PCI, percutaneous coronary intervention; eGFR, estimated glomerular filtration rate; LVEF, left ventricular ejection fraction; HbA1c, glycated hemoglobin; LDL-c, low-density lipoprotein cholesterol; HDL-c, high-density lipoprotein cholesterol; lp(a), lipoprotein (a); apo, apolipoprotein.

# Supplemental Table3. Baseline characteristics according to quintiles of LDL-c.

|  | **LDL-c, mmol/L** | | | | | **P for trend** |
| --- | --- | --- | --- | --- | --- | --- |
|  | **Q1 (n=1990)**  **<1.76** | **Q2 (n=2010)**  **1.76≤LDL-c <2.16** | **Q3 (n=1987)**  **2.16≤LDL-c <2.59** | **Q4 (n=2023)**  **2.59≤LDL-c <3.19** | **Q5 (n=1993)**  **≥3.19** |  |
| Sex (Women) | 355 (17.84) | 391 (19.45) | 455 (22.90) | 522 (25.80) | 574 (18.80) | <0.001 |
| Age, years | 58 [50, 66] | 58 [51, 66] | 59 [51, 66] | 58 [51, 65] | 58 [50, 65] | 0.036 |
| ≥65 years | 551 (27.69) | 562 (27.96) | 569 (28.64) | 530 (26.20) | 512 (25.69) | 0.067 |
| BMI, kg/m^2^ | 25.71 [23.79, 27.68] | 25.83 [23.88, 27.76] | 25.86 [23.80, 27.76] | 25.95 [24.00, 27.78] | 25.95 [24.03, 28.09] | <0.001 |
| Obesity | 428 (21.51) | 475 (23.63) | 467 (23.50) | 485 (23.97) | 525 (26.34) | 0.011 |
| Admission presentation |  |  |  |  |  |  |
| AMI | 317 (15.93) | 339 (16.87) | 370 (18.62) | 433 (21.40) | 374 (18.77) | <0.001 |
| Unstable angina | 840 (42.21) | 836 (41.59) | 814 (40.97) | 835 (41.28) | 863 (43.30) | <0.001 |
| CCS | 833 (41.86) | 835 (41.54) | 803 (40.41) | 755 (37.32) | 756 (37.93) | <0.001 |
| Smoking history | 1210 (60.80) | 1195 (59.45) | 1158 (58.28) | 1165 (57.59) | 1092 (54.79) | <0.001 |
| Diabetes | 827 (41.56) | 819 (40.75) | 788 (39.66) | 773 (38.21) | 781 (39.19) | 0.035 |
| Oral antidiabetic agents | 423 (51.15) | 355 (43.45) | 334 (42.39) | 315 (40.75) | 260 (33.29) | <0.001 |
| Insulin | 227 (27.45) | 217 (26.50) | 200 (25.38) | 211 (27.30) | 199 (25.48) | 0.518 |
| Hypertension | 1387 (69.70) | 1393 (69.30) | 1406 (70.76) | 1391 (68.76) | 1364 (68.44) | 0.347 |
| Dyslipidemia | 1471 (73.92) | 1463 (72.79) | 1476 (74.28) | 1491 (73.70) | 1644 (82.490 | <0.001 |
| Peripheral artery disease | 59 (2.970) | 58 (2.89) | 60 (3.02) | 52 (2.57) | 32 (1.61) | 0.007 |
| COPD | 45 (2.26) | 52 (2.59) | 45 (2.27) | 51 (2.52) | 39 (1.96) | 0.527 |
| Prior myocardial infarction | 460 (23.12) | 433 (21.54) | 370 (18.62) | 304 (15.03) | 301 (15.10) | <0.001 |
| Prior stroke | 232 (11.66) | 225 (11.19) | 219 (11.02) | 218 (10.78) | 186 (9.33) | 0.021 |
| Prior PCI | 548 (27.54) | 507 (25.22) | 486 (24.46) | 388 (19.18) | 369 (18.52) | <0.001 |
| eGFR, ml/min/1.73m^2^ | 119.04 [103.41, 133.69] | 118.10 [103.16, 133.68] | 118.60 [102.61, 134.57] | 118.74 [13.42, 134.26] | 117.14 [101.83, 132.91] | 0.363 |
| LVEF, % | 63.50 [60, 67] | 63.90 [60, 67] | 63.50 [60, 67] | 63.50 [60, 67] | 63.50 [60, 67] | 0.272 |
| HbA1c, % | 6.20 [5.80, 6.90] | 6.20 [5.90, 6.90] | 6.20 [5.90, 6.90] | 6.20 [5.90, 6.90] | 6.20 [5.90, 7.00] | <0.001 |
| Fasting glucose, mmol/L | 5.31 [4.84, 6.38] | 5.49 [4.92, 6.59] | 5.48 [4.91, 6.59] | 5.55 [4.99, 6.80] | 5.63 [5.05, 6.87] | <0.001 |
| Total cholesterol, mmol/L | 3.01 [2.73, 3.26] | 3.54 [3.34, 3.78] | 4.02 [3.80, 4.24] | 4.56 [4.32, 4.83] | 5.57 [5.17, 6.12] | <0.001 |
| Total triglyceride, mmol/L | 1.28 [0.96, 1.80] | 1.40 [1.06, 1.92] | 1.53 [1.15, 2.06] | 1.65 [1.25, 2.25] | 1.78 [1.37, 2.34] | <0.001 |
| LDL-c, mmol/L | 1.50 [1.31, 1.63] | 1.96 [1.86, 2.06] | 2.35 [2.25, 2.46] | 2.84 [2.71, 3.01] | 3.69 [3.41, 4.18] | <0.001 |
| non-HDL-c, mmol/L | 2.02 [1.80, 2.25] | 2.53 [2.35, 2.73] | 2.97 [2.78, 3.18] | 3.51 [3.30, 3.76] | 4.47 [4.09, 5.00] | <0.001 |
| HDL-c, mmol/L | 0.92 [0.78, 1.12] | 0.97 [0.82, 1.14] | 1.00 [0.84,1.18] | 1.00 [0.86, 1.19] | 1.07 [0.91, 1.25] | <0.001 |
| Lp(a), mg/dL | 12.39 [5.61, 29.76] | 17.10 [7.05, 37.02] | 19.74 [8.66, 41.97] | 20.21 [8.58, 45.03] | 23.96 [10.44, 49.83] | <0.001 |
| ApoB, g/L | 0.58 [0.52, 0.65] | 0.69 [0.64, 0.76] | 0.79 [0.73, 0.86] | 0.91 [0.83, 0.99] | 1.13 [1.01, 1.28] | <0.001 |
| ApoA-1, g/L | 1.25 [1.13, 1.40] | 1.30 [1.17, 1.45] | 1.32 [1.18, 1.48] | 1.33 [1.19, 1.50] | 1.38 [1.23, 1.56] | <0.001 |
| Non-HDL-c/HDL-c | 2.16 [1.70, 2.72] | 2.63 [2.12, 3.24] | 2.95 [2.41, 3.68] | 3.53 [2.86, 4.26] | 4.29 [3.51, 5.20] | <0.001 |
| ApoB/apoA-1 | 0.46 [0.39, 0.54] | 0.54 [0.46, 0.63] | 0.60 [0.52, 0.70] | 0.69 [0.58, 0.79] | 0.82 [0.70,0.98] | <0.001 |

Values are presented as median [interquartile range] or number (%).

LDL-c, low-density lipoprotein cholesterol; Q, quintile; BMI, body mass index; AMI, acute myocardial infarction; CCS, chronic coronary syndrome; COPD, chronic obstructive pulmonary disease; PCI, percutaneous coronary intervention; eGFR, estimated glomerular filtration rate; LVEF, left ventricular ejection fraction; HbA1c, glycated hemoglobin; HDL-c, high-density lipoprotein cholesterol; lp(a), lipoprotein (a); apo, apolipoprotein.

# Supplemental Table4. Baseline characteristics according to quintiles of non-HDL-c.

|  | **Non-HDL-c, mmol/L** | | | | | **P for trend** |
| --- | --- | --- | --- | --- | --- | --- |
|  | **Q1 (n=2017)**  **<2.30** | **Q2 (n=1972)**  **2.30≤non-HDL-c<2.78** | **Q3 (n=2030)**  **2.78≤non-HDL-c <3.28** | **Q4 (n=1980)**  **3.28≤non-HDL-c <3.96** | **Q5 (n=2004)**  **≥3.96** |  |
| Sex (Women) | 352(17.45) | 384 (19.47) | 501 (24.68) | 493 (24.90) | 567 (28.29) | <0.001 |
| Age, years | 60 [52, 67] | 59 [50, 66] | 58 [51, 65] | 58 [50, 65] | 57 [50, 64] | <0.001 |
| ≥65 years | 630 (31.24) | 568 (28.80) | 552 (27.19) | 507 (25.61) | 467 (23.30) | <0.001 |
| BMI, kg/m^2^ | 25.39 [23.53, 27.58] | 25.83 [23.74, 27.76] | 25.86 [23.88, 27.78] | 25.95 [24.02, 27.76] | 26.12 [24.22, 28.37] | <0.001 |
| Obesity | 390 (19.34) | 473 (23.99) | 492 (24.24) | 461 (23.28) | 564 (28.14) | <0.001 |
| Admission presentation |  |  |  |  |  |  |
| AMI | 288 (14.28) | 324 (16.43) | 409 (20.14) | 432 (21.82) | 380 (18.96) | <0.001 |
| Unstable angina | 846 (41.94) | 847 (42.95) | 842 (41.48) | 805 (40.66) | 848 (42.32) | <0.001 |
| CCS | 883 (43.78) | 801 (40.62) | 779 (38.37) | 743 (37.53) | 776 (38.72) | <0.001 |
| Smoking history | 1198 (59.40) | 1156 (58.62) | 1170 (57.63) | 1169 (59.04) | 1127 (56.24) | 0.090 |
| Diabetes | 791 (39.22) | 796 (40.37) | 815 (40.15) | 742 (37.48) | 844 (42.12) | 0.393 |
| Oral antidiabetic agents | 412 (52.09) | 354 (44.47) | 351 (43.07) | 278 (37.47) | 292 (34.60) | <0.001 |
| Insulin | 217 (27.43) | 202 (25.38) | 221 (27.12) | 200 (26.95) | 314 (25.36) | 0.583 |
| Hypertension | 1401 (69.46) | 1380 (69.98) | 1411 (69.51) | 1371 (69.24) | 1378 (68.76) | 0.515 |
| Dyslipidemia | 1356 (67.23) | 1437 (72.87) | 1482 (73.01) | 1509 (76.21) | 1761 (87.87) | <0.001 |
| Peripheral artery disease | 58 (2.88) | 59 (2.99) | 54 (2.66) | 51 (2.58) | 39 (1.95) | 0.044 |
| COPD | 36 (1.79) | 66 (3.35) | 50 (2.46) | 41 (2.07) | 39 (1.95) | 0.383 |
| Prior myocardial infarction | 468 (23.20) | 424 (21.55) | 360 (17.73) | 310 (15.66) | 305 (15.22) | <0.001 |
| Prior stroke | 231 (11.45) | 241 (12.22) | 213 (10.49) | 213 (10.76) | 182 (9.08) | 0.005 |
| Prior PCI | 571 (28.31) | 500 (25.36) | 467 (23.01) | 388 (19.60) | 372 (18.56) | <0.001 |
| eGFR, ml/min/1.73m^2^ | 119.04 [103.52, 134.15] | 118.40 [103.55, 134.00] | 118.48 [102.42, 133.81] | 118.51 [103.05, 133.89] | 117.17 [101.77, 133.13] | <0.001 |
| LVEF, % | 64 [60, 67.5] | 64 [60, 67] | 64 [60, 67] | 63 [60, 67] | 64 [60,67] | 0.126 |
| HbA1c, % | 6.20 [5.80, 6.70] | 6.20 [5.80, 6.80] | 6.20 [5.80, 7.00] | 6.20 [5.90, 7.00] | 6.30 [5.90, 7.20] | 0.013 |
| Fasting glucose, mmol/L | 5.30 [4.82, 6.30] | 5.44 [4.91, 6.48] | 5.49 [4.92, 6.66] | 5.55 [4.98, 6.79] | 5.69 [5.08, 7.05] | <0.001 |
| Total cholesterol, mmol/L | 3.00 [2.74, 3.24] | 3.54 [3.36, 3.74] | 4.02 [3.83, 4.23] | 4.58 [4.38, 4.82] | 5.64 [5.25, 6.15] | <0.001 |
| Total triglyceride, mmol/L | 1.11 [0.87, 1.44] | 1.37 [1.07, 1.77] | 1.56 [1.21, 2.02] | 1.79 [1.35, 2.40] | 2.08 [1.56, 2.91] | <0.001 |
| LDL-c, mmol/L | 1.55 [1.33, 1.72] | 2.00 [1.84, 2.15] | 2.39 [2.22, 2.57] | 2.87 [2.65, 3.10] | 3.67 [3.35, 4.18] | <0.001 |
| non-HDL-c, mmol/L | 2.01 [1.80, 2.18] | 2.55 [2.43, 2.66] | 3.01 [2.89, 3.14] | 3.58 [3.43, 3.75] | 4.54 [4.22, 5.6] | <0.001 |
| HDL-c, mmol/L | 0.99 [0.83, 1.20] | 0.98 [0.84, 1.16] | 0.99 [0.83, 1.17] | 0.99 [0.83, 1.16] | 1.02 [0.87, 1.19] | <0.001 |
| Lp(a), mg/dL | 13.85 [6.3231.79] | 16.98 [7.09, 37.27] | 19.70 [8.70, 42.73] | 20.74 [8.46, 43.69] | 21.68 [9.23, 47.64] | <0.001 |
| ApoB, g/L | 0.57 [0.52, 0.63] | 0.69 [0.64, 0.75] | 0.79 [0.74, 0.86] | 0.92 [0.85, 1.00] | 1.13 [1.02, 1.29] | <0.001 |
| ApoA-1, g/L | 1.25 [1.13, 1.42] | 1.30 [1.17, 1.45] | 1.31 [1.17, 1.48] | 1.33 [1.19, 1.49] | 1.37 [1.23, 1.55] | <0.001 |
| Non-HDL-c/HDL-c | 1.97 [1.59, 2.40] | 2.58 [2.19, 3.05] | 3.04 [2.58, 3.63] | 3.66 [3.06, 4.33] | 4.56 [3.87, 5.54] | <0.001 |
| ApoB/apoA-1 | 0.45 [0.39, 0.53] | 0.53 [0.46, 0.61] | 0.61 [0.53, 0.70] | 0.70 [0.60, 0.80] | 0.83 [0.71, 0.98] | <0.001 |

Values are presented as median [interquartile range] or number (%).

HDL-c, high-density lipoprotein cholesterol; Q, quintile; BMI, body mass index; AMI, acute myocardial infarction; CCS, chronic coronary syndrome; COPD, chronic obstructive pulmonary disease; PCI, percutaneous coronary intervention; eGFR, estimated glomerular filtration rate; LVEF, left ventricular ejection fraction; HbA1c, glycated hemoglobin; LDL-c, low-density lipoprotein cholesterol; lp(a), lipoprotein (a); apo, apolipoprotein.

# Supplemental Table5. Baseline characteristics according to quintiles of lp(a).

|  | **Lp(a), mg/dL** | | | | | **P for trend** |
| --- | --- | --- | --- | --- | --- | --- |
|  | **Q1 (n=2001)**  **<6.27** | **Q2 (n=2000)**  **6.27≤lp(a)<13.08** | **Q3 (n=2001)**  **13.08≤lp(a)<25.21** | **Q4 (n=2000)**  **25.21≤lp(a)<48.42** | **Q5 (n=2001)**  **≥48.42** |  |
| Sex (Women) | 359 (17.94) | 454 (22.70) | 442 (22.09) | 444 (22.20) | 598 (29.89) | <0.001 |
| Age, years | 57 [50, 64] | 58 [50, 65] | 58 [50, 65] | 59 [51, 66] | 59 [52, 66] | <0.001 |
| ≥65 years | 480 (23.99) | 544 (27.20) | 543 (27.14) | 592 (29.60) | 565 (28.24) | <0.001 |
| BMI, kg/m^2^ | 25.95 [24.03, 28.01] | 25.95 [24.03, 27.92] | 25.91 [23.88, 27.78] | 25.88 [23.88, 27.78] | 25.51 [23.63, 27.68] | 0.010 |
| Obesity | 506 (25.29) | 495 (24.75) | 487 (24.34) | 484 (24.20) | 408 (20.39) | 0.002 |
| Admission presentation |  |  |  |  |  |  |
| AMI | 303 (15.15) | 352 (17.60) | 374 (18.69) | 403 (20.15) | 401 (20.04) | <0.001 |
| Unstable angina | 874 (43.68) | 848 (42.40) | 866 (43.28) | 781 (39.05) | 819 (40.93) | <0.001 |
| CCS | 824 (42.28) | 800 (40.00) | 761 (38.03) | 816 (40.80) | 781 (39.03) | <0.001 |
| Smoking history | 1237 (61.82) | 1174 (58.70) | 1172 (58.57) | 1143 (57.15) | 1094 (54.67) | <0.001 |
| Diabetes | 870 (43.78) | 794 (39.70) | 777 (38.83) | 770 (38.50) | 777 (38.83) | 0.002 |
| Oral antidiabetic agents | 381 (43.79) | 310 (39.04) | 366 (47.10) | 303 (39.35) | 327 (42.09) | 0.557 |
| Insulin | 222 (25.52) | 226 (28.46) | 182 (23.42) | 222 (28.83) | 202 (26.00) | 0.773 |
| Hypertension | 1405 (70.22) | 1407 (70.35) | 1369 (68.42) | 1370 (68.50) | 1390 (69.47) | 0.304 |
| Dyslipidemia | 1567 ()78.31 | 1505 (75.25) | 1486 (74.26) | 1481 (74.05) | 1506 (75.26) | 0.017 |
| Peripheral artery disease | 48 (2.40) | 58 (2.90) | 55 (2.75) | 48 (2.40) | 52 (2.60) | 0.929 |
| COPD | 38 (1.90) | 35 (175) | 50 (2.50) | 52 (2.60) | 57 (2.85) | 0.010 |
| Prior myocardial infarction | 367 (18.34) | 381 (19.05) | 358 (17.89) | 370 (18.50) | 392 (19.59) | 0.479 |
| Prior stroke | 214 (10.70) | 194 (9.70) | 201 (10.05) | 230 (11.50) | 241 (12.04) | 0.040 |
| Prior PCI | 462 (23.09) | 473 (23.65) | 440 (21.99) | 426 (21.30) | 497 (24.84) | 0.699 |
| eGFR, ml/min/1.73m^2^ | 119.62 [105.20, 134.45] | 118.20 [102.99, 133.24] | 118.50 [102.23, 134.44] | 117.80 [101.89, 133.46] | 117.23 [101.47, 133.49] | 0.001 |
| LVEF, % | 64 [60, 67] | 64 [60, 67] | 64 [60, 67] | 63 [60, 67] | 64 [60, 67] | 0.011 |
| HbA1c, % | 6.20 [5.8, 7.10] | 6.20 [5.80, 6.90] | 6.20 [5.80, 6.90] | 6.20 [5.80, 6.90] | 6.20 [5.80, 6.90] | 0.272 |
| Fasting glucose, mmol/L | 5.59 [5.00, 6.88] | 5.51 [4.98, 6.66] | 5.45 [4.92, 6.67] | 5.47 [4.92, 6.58] | 5.45 [4.88, 6.48] | 0.001 |
| Total cholesterol, mmol/L | 3.89 [3.30, 4.64] | 3.93 [3.36, 4.69] | 4.06 [3.43, 4.79] | 4.13 [3.53, 4.8] | 4.27 [3.64, 5.02] | <0.001 |
| Total triglyceride, mmol/L | 1.64 [1.18, 2.43] | 1.55 [1.13, 2.18] | 1.50 [1.14, 2.01] | 1.50 [1.12, 1.01] | 1.49 [1.15, 1.94] | <0.001 |
| LDL-c, mmol/L | 2.17 [1.70, 2.78] | 2.26 [1.78. 2.89] | 2.38 [1.89, 3.03] | 2.42 [1.94, 3.09] | 2.56 [2.05, 3.22] | <0.001 |
| non-HDL-c, mmol/L | 2.84 [2.31, 3.61] | 2.91 [2.34, 3.65] | 3.03 [2.43, 3.75] | 3.08 [2.49, 3.80] | 3.18 [2.58, 3.93] | <0.001 |
| HDL-c, mmol/L | 0.98 [0.83, 1.15] | 0.98 [0.83, 1,16] | 0.99 [0.84, 1.17] | 0.99 [0.84, 1.17] | 1.04 [0.87, 1.23] | <0.001 |
| Lp(a), mg/dL | 3.60 [2.14, 4.89] | 9.40 [7.80, 11.19] | 18.31 [15.48, 21.40] | 34.50 [29.55, 40.86] | 73.69 [59.24, 92.73] | <0.001 |
| ApoB, g/L | 0.74 [0.62, 0.91] | 0.76 [0.63, 0.92] | 0.80 [0.67, 0.96] | 0.82 [0.68, 1.00] | 0.87 [0.74, 1.05] | <0.001 |
| ApoA-1, g/L | 1.32 [1.18, 1.48] | 1.31 [1.18, 1.48] | 1.30 [1.17, 1.47] | 1.30 [1.17, 1.47] | 1.34 [1.18, 1.52] | 0.606 |
| Non-HDL-c/HDL-c | 2.94 [2.23, 3.93] | 3.01 [2.24, 3.95] | 3.07 [2.35,4.01] | 3.12 [2.37, 4.08] | 3.09 [2.40, 4.02] | <0.001 |
| ApoB/apoA-1 | 0.57 [0.46,0.70] | 0.58 [0.47, 0.71] | 0.61 [0.50, 0.75] | 0.63 [0.51, 0.78] | 0.67 [0.54, 0.81] | <0.001 |

Values are presented as median [interquartile range] or number (%).

Lp(a), lipoprotein (a); Q, quintile; BMI, body mass index; AMI, acute myocardial infarction; CCS, chronic coronary syndrome; COPD, chronic obstructive pulmonary disease; PCI, percutaneous coronary intervention; eGFR, estimated glomerular filtration rate; LVEF, left ventricular ejection fraction; HbA1c, glycated hemoglobin; LDL-c, low-density lipoprotein cholesterol; HDL-c, high-density lipoprotein cholesterol; apo, apolipoprotein.

# Supplemental Table6. Baseline characteristics according to quintiles of apoB.

|  | **ApoB, g/L** | | | | | **P for trend** |
| --- | --- | --- | --- | --- | --- | --- |
|  | **Q1 (n=1997)**  **<0.63** | **Q2 (n=2026)**  **0.63≤apoB<0.75** | **Q3 (n=1900)**  **0.75≤apoB<0.86** | **Q4 (n=2065)**  **0.86≤apoB<1.02** | **Q5 (n=2015)**  **≥1.02** |  |
| Sex (Women) | 395 (19.78) | 391 (19.30) | 432 (22.74) | 524 (25.38) | 555 (27.54) | <0.001 |
| Age, years | 60 [52. 67] | 59 [51, 66] | 58 [50,65] | 58 [51, 65] | 57 [50, 64] | <0.001 |
| ≥65 years | 636 (31.85) | 590 (29.12) | 507 (26.68) | 529 (25.62) | 462 (22.93) | <0.001 |
| BMI, kg/m^2^ | 25.39 [23.44, 27.64] | 25.83 [23.83, 27.76] | 25.91 [24.06, 27.73] | 25.95 [23.88, 28.01] | 26.04 [24.22, 28.34] | <0.001 |
| Obesity | 391 (19.58) | 473 (23.35) | 437 (23.00) | 517 (25.04) | 562 (27.89) | <0.001 |
| Admission presentation |  |  |  |  |  |  |
| AMI | 291 (14.58) | 347 (17.13) | 352 (18.53) | 434 (21.02) | 409 (20.30) | <0.001 |
| Unstable angina | 869 (43.52) | 814 (40.18) | 804 (42.32) | 884 (42.81) | 817 (40.55) | <0.001 |
| CCS | 837 (41.91) | 865 (42.70) | 744 (39.16) | 747 (36.17) | 789 (39.16) | <0.001 |
| Smoking history | 1158 (57.99) | 1203 (59.38) | 1084 (57.05) | 1235 (59.81) | 1140 (56.58) | 0.496 |
| Diabetes | 791 (39.61) | 773 (38.15) | 735 (38.68) | 850 (41.16) | 839 (41.64) | 0.039 |
| Oral antidiabetic agents | 400 (50.57) | 349 (45.15) | 317 (43.13) | 331 (38.94) | 290 (34.57) | <0.001 |
| Insulin | 224 (28.32) | 179 (23.16) | 203 (27.62) | 240 (28.24) | 208 (24.79) | 0.674 |
| Hypertension | 1400 (70.11) | 1419 (70.04) | 1320 (69.47) | 1421 (68.81) | 1381 (68.54) | 0.178 |
| Dyslipidemia | 1383 (69.25) | 1455 (71.82) | 1410 (74.21) | 1578 (76.42) | 1719 (85.31) | <0.001 |
| Peripheral artery disease | 61 (3.06) | 56 (2.76) | 47 (2.47) | 58 (2.81) | 39 (1.94) | 0.052 |
| COPD | 49 (2.45) | 48 (2.37) | 44 (2.32) | 51 (2.47) | 40 (1.99) | 0.433 |
| Prior myocardial infarction | 450 (22.53) | 430 (21.22) | 343 (18.05) | 325 (15.74) | 320 (15.88) | <0.001 |
| Prior stroke | 233 (11.67) | 232 (11.45) | 220 (11.58) | 211 (10.22) | 184 (9.13) | 0.004 |
| Prior PCI | 544 (27.24) | 514 (25.37) | 427 (22.47) | 420 (20.34) | 393 (19.50) | <0.001 |
| eGFR, ml/min/1.73m^2^ | 118.72 [102.93, 133.70] | 117.81 [102.59,132.80] | 120.42 [104.62, 136.11] | 117.58 [102.90, 132.63] | 117.63 [101.58, 133.47] | 0.456 |
| LVEF, % | 64 [60, 67] | 64 [60, 67] | 64 [60, 67] | 64 [60, 67] | 64 [60, 67] | 0.011 |
| HbA1c, % | 6.20 [5.80, 6.70] | 6.20 [5.80, 6.80] | 6.20 [5.80, 7.00] | 6.20 [5.90, 7.10] | 6.30 [5.90, 7.15] | <0.001 |
| Fasting glucose, mmol/L | 5.28 [4.80, 6.32] | 5.40 [4.89, 6.38] | 5.49 [4.91, 6.67] | 5.60 [5.04, 6.81] | 5.71 [5.10,7.09] | <0.001 |
| Total cholesterol, mmol/L | 3.06 [2.76, 3.38] | 3.61 [3.33, 3.91] | 4.05 [3.74, 4.36] | 4.56 [4.21, 4.95] | 5.51 [4.99, 6.11] | <0.001 |
| Total triglyceride, mmol/L | 1.14 [0.88, 1.50] | 1.38 [1.06, 1.80] | 1.56 [1.19, 2.02] | 1.74 [1.33, 2.37] | 2.00 [1.53, 2.69] | <0.001 |
| LDL-c, mmol/L | 1.57 [1.35, 1.79] | 2.02 [1.80, 2.24] | 2.39 [2.13, 2.65] | 2.82 [2.48, 3.16] | 3.59 [3.14, 4.16] | <0.001 |
| non-HDL-c, mmol/L | 2.06 [1.82, 2.29] | 2.60 [2.37, 2.82] | 3.04 [2.76, 3.30] | 3.56 [3.23, 3.89] | 4.45 [3.96, 5.01] | <0.001 |
| HDL-c, mmol/L | 0.97 [0.82, 1.17] | 0.99 [0.83, 1.17] | 0.99 [0.84, 1.17] | 0.99 [0.83, 1.17] | 1.03 [0.88, 1.19] | <0.001 |
| Lp(a), mg/dL | 11.94 [5.73, 26.92] | 16.64 [7.05, 34.99] | 19.34 [8.60, 42.63] | 20.88 [8.74, 46.43] | 26.47 [10.78, 55.41] | <0.001 |
| ApoB, g/L | 0.57 [0.51, 0.60] | 0.69 [0.67, 0.72] | 0.80 [0.77, 0.83] | 0.93 [0.89, 0.97] | 1.16 [1.07, 1.30] | <0.001 |
| ApoA-1, g/L | 1.26 [1.13, 1.42] | 1.30 [1.17, 1.46] | 1.31 [1.17, 1.48] | 1.32 [1.19, 1.50] | 1.36 [1.22, 1.53] | <0.001 |
| Non-HDL-c/HDL-c | 2.08 [1.66, 2.58] | 2.62 [2.17, 3.20] | 3.01 [2.50, 3.71] | 3.60 [2.91, 4.33] | 4.34 [3.56, 5.31] | <0.001 |
| ApoB/apoA-1 | 0.44 [0.38, 0.49] | 0.53 [0.57, 0.59] | 0.61 [0.54, 0.68] | 0.70 [0.62, 0.78] | 0.86 [0.76, 1.00] | <0.001 |

Values are presented as median [interquartile range] or number (%).

Apo, apolipoprotein; Q, quintile; BMI, body mass index; AMI, acute myocardial infarction; CCS, chronic coronary syndrome; COPD, chronic obstructive pulmonary disease; PCI, percutaneous coronary intervention; eGFR, estimated glomerular filtration rate; LVEF, left ventricular ejection fraction; HbA1c, glycated hemoglobin; LDL-c, low-density lipoprotein cholesterol; HDL-c, high-density lipoprotein cholesterol; lp(a), lipoprotein (a).

# Supplemental Table7. Baseline characteristics according to quintiles of non-HDL-c/HDL-c.

|  | **Non-HDL-c/HDL-c, mmol/L** | | | | | **P for trend** |
| --- | --- | --- | --- | --- | --- | --- |
|  | **Q1 (n=2003)**  **<2.17** | **Q2 (n=2004)**  **2.17≤ratio<2.74** | **Q3 (n=1998)**  **2.74≤ratio<3.39** | **Q4 (n=1998)**  **3.39≤ratio<4.27** | **Q5 (n=2000)**  **≥4.27** |  |
| Sex (Women) | 590 (29.46) | 472 (23.55) | 451 (22.57) | 436 (21.82) | 348 (17.40) | <0.001 |
| Age, years | 61 [55,68] | 59 [53, 66] | 58 [50, 65] | 58 [50, 65] | 55 [48, 62] | <0.001 |
| ≥65 years | 741 (37.00) | 591 (29.49) | 527 (26.38) | 502 (25.13) | 363 (18.15) | <0.001 |
| BMI, kg/m^2^ | 24.91 [22.83, 26.89] | 25.76 [23.72, 27.72] | 25.95 [24.16, 27.77] | 25.95 [24.09, 28.08] | 26.49 [24.51, 28.52] | <0.001 |
| Obesity | 316 (15.78) | 464 (23.15) | 475 (23.77) | 522 (26.13) | 603 (30.15) | <0.001 |
| Admission presentation |  |  |  |  |  |  |
| AMI | 205 (10.24) | 275 (13.72) | 403 (20.18) | 450 (22.58) | 499 (24.95) | <0.001 |
| Unstable angina | 918 (45.83) | 870 (43.41) | 785 (39.29) | 804 (40.24) | 811 (40.55) | <0.001 |
| CCS | 880 (43.93) | 859 (42.86) | 810 (40.54) | 743 (37.19) | 690 (34.50) | <0.001 |
| Smoking history | 1031 (51.47) | 1099 (54.84) | 1142 (57.16) | 1206 (60.36) | 1342 (67.10) | <0.001 |
| Diabetes | 708 (35.35) | 777 (38.77) | 813 (40.69) | 842 (42.14) | 848 (42.40) | <0.001 |
| Oral antidiabetic agents | 344 (48.59) | 356 (48.82) | 354 (43.54) | 332 (39.43) | 301 (35.50) | <0.001 |
| Insulin | 197 (27.83) | 178 (22.91) | 211 (25.95) | 247 (29.34) | 221 (26.06) | 0.508 |
| Hypertension | 1416 (70.69) | 1412 (70.46) | 1371 (68.62) | 1397 (69.92) | 1345 (67.25) | 0.023 |
| Dyslipidemia | 1282 (64.00) | 1375 (68.61) | 1477 (73.92) | 1605 (80.33) | 1806 (90.30)) | <0.001 |
| Peripheral artery disease | 62 (3.10) | 59 (2.94) | 50 (2.50) | 44 (2.20) | 46 (2.30) | 0.039 |
| COPD | 50 (2.50) | 49 (2.45) | 39 (1.95) | 54 (2.70) | 40 (2.00) | 0.489 |
| Prior myocardial infarction | 379 (18.92) | 420 (20.96) | 396 (19.82) | 331 (16.57) | 342 (17.10) | 0.004 |
| Prior stroke | 236 (11.78) | 221 (11.03) | 207 (10.36) | 219 (10.96) | 197 (9.85) | 0.073 |
| Prior PCI | 532 (25.56) | 491 (24.50) | 471 (23.57) | 408 (20.42) | 396 (19.80) | <0.001 |
| eGFR, ml/min/1.73m^2^ | 117.92 [102.53, 133.12] | 118.50 [103.72, 133.48] | 118.18 [102.80, 133.59] | 117.97 [101.43, 133.83] | 118.56 [103.71, 134.88] | 0.454 |
| LVEF, % | 64 [60, 68] | 64 [60.68] | 64 [60,67] | 63 [60, 67] | 63 [59, 67] | <0.001 |
| HbA1c, % | 6.10 [5.80, 6.60] | 6.20 [5.80, 6.80] | 6.20 [5.90, 6.98] | 6.20 [5.90, 7.20] | 6.30 [5.90,7.30] | <0.001 |
| Fasting glucose, mmol/L | 5.33 [4.83, 6.26] | 5.42 [4.95,6.45] | 5.51 [4.95, 6.70] | 5.58 [4.95, 6.87] | 6.64 [5.01, 7.07] | <0.001 |
| Total cholesterol, mmol/L | 3.43 [3.00, 3.93] | 3.69 [3.23, 4.23] | 4.00 [3.52, 4.59] | 4.40 [3.83, 5.04] | 5.06 [4.34, 5.87] | <0.001 |
| Total triglyceride, mmol/L | 1.06 [0.84, 1.36] | 1.33 [1.06, 1.69] | 1.56 [1.23, 2.02] | 1.77 [1.39, 2.33] | 2.23 [1.67, 3.08] | <0.001 |
| LDL-c, mmol/L | 1.75 [1.44, 2.11] | 2.10 [1.75, 2.50] | 2.37 [1.98, 2.88] | 2.74 [2.26, 3.26] | 3.21 [2.61, 3.89] | <0.001 |
| non-HDL-c, mmol/L | 2.15 [1.86, 2.49] | 2.62 [2.30, 3.02] | 3.00 [2.64, 3.46] | 3.47 [3.03, 3.99] | 4.24 [3.62, 4.95] | <0.001 |
| HDL-c, mmol/L | 1.25 [1.08, 1.48] | 1.07 [0.93, 1.22] | 0.99 [0.87, 1.13] | 0.92 [0.80, 1.05] | 0.81 [0.70, 0.93] | <0.001 |
| Lp(a), mg/dL | 15.14 [6.79, 37.01] | 18.59 [7.88, 41.12] | 19.61 [8.07, 41,69] | 19.28 [8.21, 42.45] | 19.22 [8.39, 40.84] | 0.001 |
| ApoB, g/L | 0.62 [0.54, 0.71] | 0.72 [0.63, 0.83] | 0.80 [0.70, 0.93] | 0.89 [0.78, 1.03] | 1.03 [0.89, 1.22] | <0.001 |
| ApoA-1, g/L | 1.45 [1.29, 1.67] | 1.35 [1.22, 1.52] | 1.32 [1.19, 1.46] | 1.27 [1.15, 1.40] | 1.21 [1.10, 1.33] | <0.001 |
| Non-HDL-c/HDL-c | 1.79 [1.52, 2.00] | 2.46 [2.33, 2.61] | 3.05 [2.89, 3.22] | 3.78 [3.58, 4.00] | 5.03 [4.58, 5.77] | <0.001 |
| ApoB/apoA-1 | 0.43 [0.37, 0.49] | 0.53 [0.47, 0.59] | 0.61 [0.54, 0.69] | 0.70 [0.63, 0,79] | 0.85 [0.74, 0.99] | <0.001 |

Values are presented as median [interquartile range] or number (%).

HDL-c, high-density lipoprotein cholesterol; Q, quintile; BMI, body mass index; AMI, acute myocardial infarction; CCS, chronic coronary syndrome; COPD, chronic obstructive pulmonary disease; PCI, percutaneous coronary intervention; eGFR, estimated glomerular filtration rate; LVEF, left ventricular ejection fraction; HbA1c, glycated hemoglobin; LDL-c, low-density lipoprotein cholesterol; lp(a), lipoprotein (a); apo, apolipoprotein.

# Supplemental Table8. Baseline characteristics according to quintiles of apoB/apoA-1.

|  | **ApoB/apoA-1** | | | | | **P for trend** |
| --- | --- | --- | --- | --- | --- | --- |
|  | **Q1 (n=2012)**  **<0.47** | **Q2 (n=1978)**  **0.47≤ratio<0.57** | **Q3 (n=2008)**  **0.57≤ratio<0.66** | **Q4 (n=2007)**  **0.66≤ratio<0.79** | **Q5 (n=1998)**  **≥0.79** |  |
| Sex (Women) | 616 (30.62) | 440 (22.25) | 452 (22.51) | 422 (21.03) | 367 (18.37) | <0.001 |
| Age, years | 61 [54, 68] | 59 [52, 66] | 59 [51, 65] | 57 [50, 64] | 56 [48, 63] | <0.001 |
| ≥65 years | 707 (35.14) | 598 (30.23) | 537 (26.74) | 489 24.37( | 393 (19.67) | <0.001 |
| BMI, kg/m^2^ | 25.25 [23.01, 27.34] | 25.71 [23.72, 23.68] | 25.95 [24.03, 27.78] | 25.95 [24.17, 27.91] | 26.26 [24.22, 28.41] | <0.001 |
| Obesity | 365 (18.14) | 432 (21.84) | 490 (24.40) | 500 (24.91) | 593 (29.68) | <0.001 |
| Admission presentation |  |  |  |  |  |  |
| AMI | 228 (11.33) | 263 (13.30) | 354 (17.63) | 458 (22.82) | 530 (26.53) | <0.001 |
| Unstable angina | 902 (44.83) | 872 (44.09) | 837 (41.68) | 789 (39.31) | 788 (39.44) | <0.001 |
| CCS | 882 (43.84) | 843 (42.62) | 817 (40.69) | 760 (37.87) | 680 (34.03) | <0.001 |
| Smoking history | 1029 (51.14) | 1102 (55.71) | 1165 (58.02) | 1245 (62.03) | 1279 (64.01) | <0.001 |
| Diabetes | 737 (36.63) | 771 (38.98) | 795 (39.59) | 822 (40.96) | 863 (43.19) | <0.001 |
| Oral antidiabetic agents | 364 (49.39) | 332 (43.06) | 343 (43.15) | 326 (39.66) | 322 (37.31) | <0.001 |
| Insulin | 187 (25.37) | 213 (27.63) | 204 (25.66) | 232 (28.22) | 218 (25.26) | 0.981 |
| Hypertension | 1432 (71.17) | 1367 (69.11) | 1425 (70.97) | 1398 (69.66) | 1319 (66.02) | 0.003 |
| Dyslipidemia | 1360 (67.59) | 1418 (71.69) | 1504 (74.90) | 1584 (78.92) | 1679 (84.03) | <0.001 |
| Peripheral artery disease | 57 (2.83) | 60 (3.03) | 57 (2.84) | 44 (2.19) | 43 (2.115) | 0.051 |
| COPD | 51 (2.54) | 45 (2.28) | 49 (2.44) | 39 (1.94) | 48 (2.40) | 0.572 |
| Prior myocardial infarction | 386 (19.19) | 406 (20.53) | 402 (20.02) | 342 (17.04) | 332 (16.62) | 0.002 |
| Prior stroke | 236 (11.73) | 196 (9.91) | 233 (11.60) | 227 (11.31) | 188 (9.41) | 0.138 |
| Prior PCI | 535 (26.59) | 454 (22.95) | 476 (23.71) | 441 (21.97) | 392 (19.62) | <0.001 |
| eGFR, ml/min/1.73m^2^ | 118.22 [102.43, 133.61] | 117.88 [103.44, 132.59] | 118.51 [102.70, 134,24] | 119.03 [103.39, 134.75] | 117.93 [102.22, 133.94] | 0.481 |
| LVEF, % | 64 [60, 68] | 64 [60, 68] | 64 [60, 67] | 64 [60, 67] | 63 [59, 67] | <0.001 |
| HbA1c, % | 6.20 [5.80, 6.70] | 6.20 [5.80, 6.80] | 6.20 [5.90, 6.90] | 6.20 [5.90, 7.10] | 6.30 [5.90, 7.30] | <0.001 |
| Fasting glucose, mmol/L | 5.36 [4.86, 6.26] | 5.43 [4.91, 6.46] | 5.46 [4.93, 6.56] | 5.55 [4.98, 6.90] | 5.69 [5.04, 7.16] | <0.001 |
| Total cholesterol, mmol/L | 3.45 [3.00 , 3.94] | 3.69 [3.22, 4.25] | 3.98 [3.49, 4.57] | 4.35 [3.82, 4.98] | 5.08 [4.41, 5.85] | <0.001 |
| Total triglyceride, mmol/L | 1.17 [0.90, 1.55] | 1.39 [1.07, 1.83] | 1.53 [1.16, 2.08] | 1.70 [1.31, 2.29] | 1.95 [1.50, 2.63] | <0.001 |
| LDL-c, mmol/L | 1.73 [1.42, 2.08] | 2.05 [1.17, 2.45] | 2.33 [1.97, 2.78] | 2.69 [2.26, 3.18] | 3.33 2.78, 4.00] | <0.001 |
| non-HDL-c, mmol/L | 2.22 [1.89, 2.59] | 2.64 [2.28, 3.08] | 2.99 [2.58, 3.48] | 3.42 [2.94, 3.93] | 4.17 [3.58, 4.89] | <0.001 |
| HDL-c, mmol/L | 1.18 [0.99, 1.42] | 1.04 [0.89, 1.22] | 0.98 [0.84, 1.14] | 0.93 [0.80, 1.09] | 0.88 [0.76, 1.03] | <0.001 |
| Lp(a), mg/dL | 12.54 [5.87, 29.76] | 16.13 [7.06, 36.06] | 17.55 [7.79, 39.09] | 22.41 [8.94, 47.93] | 25.27 [10.64, 52.81] | <0.001 |
| ApoB, g/L | 0.59 [0.52, 0.67] | 0.70 [0.63, 0.78] | 0.79 [0.72, 0.88] | 0.90 [0.82, 1.01] | 1.11 [0.99, 1.29] | <0.001 |
| ApoA-1, g/L | 1.48 [1.32, 1.69] | 1.36 [1.23, 1.51] | 1.30 [1.18, 1.46] | 1.26 [1.15, 1.40] | 1.21 [1.09, 1.33] | <0.001 |
| Non-HDL-c/HDL-c | 1.88 [1.55, 2.26] | 2.57 [2.21, 2.95] | 3.05 [2.60, 3.58] | 3.69 [3.13, 4.26] | 4.68 [3.98, 5.61] | <0.001 |
| ApoB/apoA-1 | 0.41 [0.37, 0.44] | 0.52 [0.49, 0.54] | 0.61 [0.58, 0.63] | 0.72 [0.69, 0.75] | 0.90 [0.84, 1.01] | <0.001 |

Values are presented as median [interquartile range] or number (%).

Apo, apolipoprotein; Q, quintile; BMI, body mass index; AMI, acute myocardial infarction; CCS, chronic coronary syndrome; COPD, chronic obstructive pulmonary disease; PCI, percutaneous coronary intervention; eGFR, estimated glomerular filtration rate; LVEF, left ventricular ejection fraction; HbA1c, glycated hemoglobin; LDL-c, low-density lipoprotein cholesterol; HDL-c, high-density lipoprotein cholesterol; lp(a), lipoprotein (a).

# Supplemental Table9. Associations of different lipid measures as continuous variables with total occlusion by subgroups.

| **Subgroups** | **Adjusted odds ratio (95% confidence interval)** | | | | | | | |
| --- | --- | --- | --- | --- | --- | --- | --- | --- |
|  | **TC** | **TG** | **LDL-c** | **Non-HDL-c** | **Lp(a)** | **ApoB** | **Non-HDL-c**  **/HDL-c** | **ApoB/apoA-1** |
| Sex |  |  |  |  |  |  |  |  |
| Women | 1.032  (0.928, 1.148) | 1.053  (0.940, 1.179) | 1.084  (0.960, 1.225) | 1.100  (0.987, 1.227) | 1.004  (1.000, 1.008) | 1.65  (1.053, 2.587) | 1.178  (1.075, 1.291) | 3.498  (1.985, 6.162) |
| Men | 1.081  (1.026, 1.139) | 1.005  (0.955, 1.057) | 1.128  (1.061, 1.200) | 1.114  (1.055, 1.176) | 1.003  (1.001, 1.005) | 1.580  (1.262, 1.978) | 1.124  (1.081, 1.168) | 2.383  (1.840, 3.086) |
| Age |  |  |  |  |  |  |  |  |
| <65 years | 1.082  (1.026, 1.141) | 1.012  (0.963, 1.064) | 1.123  (1.056, 1.195 | 1.113  (1.054, 1.176) | 1.003  (1.001, 1.005) | 1.669  (1.329, 2.095) | 1.121  (1.077, 1.166) | 2.483  (1.906, 3.234) |
| ≥65 years | 1.042  (0.941, 1.153) | 1.033  (0.904, 1.181) | 1.112  (0.990, 1.250) | 1.112  (1.002, 1.234) | 1.006  (1.003, 1.009) | 1.424  (0.928, 2.187) | 1.179  (1.087, 1.278) | 2.858  (1.715, 4.762) |
| Diabetes |  |  |  |  |  |  |  |  |
| Yes | 1.045  (0.970, 1.125) | 1.022  (0.960, 1.089) | 1.069  (0.978, 1.168) | 1.073  (0.994, 1.159) | 1.004  (1.001, 1.007) | 1.339  (0.977, 1.836) | 1.088  (1.028, 1.152) | 1.921  (1.324, 2.787) |
| No | 1.099  (1.034, 1.167) | 1.031  (0.966, 1.101) | 1.160  (1.082, 1.243) | 1.151  (1.082, 1.225) | 1.003  (1.001, 1.005) | 1.883  (1.453, 2.440) | 1.173  (1.121, 1.228) | 3.274  (2.428, 4.416) |
| Admission presentation | |  |  |  |  |  |  |  |
| AMI | 1.103  (1.001, 1.216) | 0.936  (0.845, 1.036) | 1.165  (1.041, 1.303) | 1.141  (1.031, 1.262) | 1.000  (0.996, 1.004) | 1.359  (0.906, 2.036) | 1.114  (1.040, 1.192) | 2.040  (1.311, 3.175) |
| UA | 1.079  (0.997, 1.168) | 1.061  (0.987, 1.140) | 1.118  (1.020, 1.225) | 1.110  (1.024, 1.204) | 1.005  (1.002, 1.008) | 1.835  (1.315, 2.560) | 1.122  (1.056, 1.192) | 2.440  (1.642, 3.626) |
| CCS | 1.037  (0.957, 1.125) | 1.064  (0.987, 1.146) | 1.048  (0.952, 1.154) | 1.062  (0.977, 1.154) | 1.003  (1.000, 1.007) | 1.465  (1.035, 2.074) | 1.091  (1.025, 1.161) | 1.922  (1.260, 2.932) |

Adjusted for sex, age, body mass index, hypertension, diabetes, prior myocardial infarction, prior percutaneous coronary intervention, smoking history, and admission presentation. LDL-c, low-density lipoprotein cholesterol; HDL-c, high-density lipoprotein cholesterol; lp(a), lipoprotein (a); apo, apolipoprotein; AMI, acute myocardial infarction; UA, unstable angina; CCS, chronic coronary syndrome.

# Supplemental Table10. AUROC of each lipid measure by subgroups.

| **Subgroups** | **AUROC (95% CI)** | | | | | | | |
| --- | --- | --- | --- | --- | --- | --- | --- | --- |
|  | **TC** | **TG** | **LDL-c** | **Non-HDL-c** | **Lp(a)** | **ApoB** | **Non-HDL-c**  **/HDL-c** | **ApoB/apoA-1** |
| Sex |  |  |  |  |  |  |  |  |
| Women | 0.506  (0.460, 0.529) | 0.519  (0.485, 0.553) | 0.508  (0.474, 0.542) | 0.517  (0.483, 0.551) | 0.543  (0.511, 0.576) | 0.523  (0.489, 0.558) | 0.565  (0.532, 0.597) | 0.574  (0.541, 0.607) |
| Men | 0.518  (0.502, 0.535) | 0.520  (0.504, 0.543) | 0.526  (0.510, 0.543) | 0.532  (0.515, 0.548) | 0.526  (0.510, 0.542) | 0.533  (0.517, 0.549) | 0.556  (0.540, 0.572) | 0.563  (0.547, 0.579) |
| Age |  |  |  |  |  |  |  |  |
| <65 years | 0.509  (0.492, 0.536) | 0.518  (0.502, 0.535) | 0.519  (0.502, 0.536) | 0.526  (0.509, 0.542) | 0.518  (0.501, 0.534) | 0.531  (0.514, 0.548) | 0.559  (0.542, 0.576) | 0.569  (0.553, 0.586) |
| ≥65 years | 0.501  (0.468, 0.527) | 0.502  (0.473, 0.532) | 0.516  (0.488, 0.545) | 0.518  (0.489, 0.547) | 0.554  (0.526, 0.582) | 0.514  (0.485, 0.543) | 0.559  (0.531, 0.588) | 0.560  (0.531, 0.588) |
| Diabetes |  |  |  |  |  |  |  |  |
| Yes | 0.503  (0.480, 0.526) | 0.506  (0.483, 0.529) | 0.510  (0.487, 0.533) | 0.517  (0.494, 0.539) | 0.528  (0.505, 0.551) | 0.519  (0.496, 0.543) | 0.546  (0.523, 0.569) | 0.555  (0.532, 0.577) |
| No | 0.510  (0.490, 0.529) | 0.527  (0.508, 0.544) | 0.525  (0.506, 0.512) | 0.530  (0.512, 0.549) | 0.525  (0.506, 0.543) | 0.533  (0.514, 0.552) | 0.571  (0.552, 0.589) | 0.577  (0.559, 0.596) |
| Admission presentation | |  |  |  |  |  |  |  |
| AMI | 0.530  (0.502, 0.558) | 0.541  (0.513, 0.568) | 0.543  (0.516, 0.571) | 0.536  (0.509, 0.564) | 0.510  (0.494, 0.549) | 0.521  (0.494, 0.549) | 0.541  (0.513, 0.569) | 0.552  (0.524, 0.579) |
| UA | 0.510  (0.484, 0.535) | 0.535  (0.511, 0.559) | 0.518  (0.493, 0.543) | 0.524  (0.499, 0.549) | 0.523  (0.497, 0.548) | 0.532  (0.506, 0.557) | 0.550  (0.525, 0.574) | 0.554  (0.529, 0.579) |
| CCS | 0.517  (0.492, 0.543) | 0.534  (0.509, 0.560) | 0.514  (0.489, 0.539) | 0.517  (0.471, 0.522) | 0.522  (0.497, 0.547) | 0.510  (0.485, 0.535) | 0.537  (0.513, 0.562) | 0.542  (0.518, 0.567) |

AUROC, area under the receiver operating characteristic curve; CI, confidence interval; apo, apolipoprotein; LDL-c, low-density lipoprotein cholesterol; HDL-c, high-density lipoprotein cholesterol; lp(a), lipoprotein (a); apo, apolipoprotein; AMI, acute elevation myocardial infarction; UA, unstable angina; CCS, chronic coronary syndrome.

# Supplemental Table11. Associations of different lipid measures with total occlusion in patients with acute coronary syndrome.

|  | **Number (%)** | **Model 1** | | **Model 2** | | **Model 3** | |
| --- | --- | --- | --- | --- | --- | --- | --- |
|  |  | **OR (95%CI)** | **p** | **OR (95%CI)** | **p** | **OR (95%CI)** | **p** |
| **TC, mmol/L** |  |  |  |  |  |  |  |
| Per SD increase | — | 1.078 (1.018, 1.141) | 0.010 | 1.088 (1.027, 1.153) | 0.004 | 1.105 (1.040, 1.174) | 0.001 |
| Q1: <3.31 | 246 (19.26) | 1.0 (Ref.) | — | 1.0 (Ref.) | — | 1.0 (Ref.) | — |
| Q2: 3.31≤TC<3.81 | 221 (17.31) | 0.846 (0.691, 1.037) | 0.108 | 0.852 (0.695, 1.044) | 0.123 | 0.855 (0.692, 1.056) | 0.145 |
| Q3: 3.81≤TC<4.32 | 261 (20.44) | 0.961 (0.790, 1.170) | 0.693 | 0.978 (0.803, 1.191) | 0.826 | 0.958 (0.780, 1.176) | 0.682 |
| Q4: 4.32≤TC<5.03 | 264 (20.67) | 0.968 (0.796, 1.178) | 0.748 | 0.989 (0.812, 1.204) | 0.914 | 0.958 (0.780, 1.176) | 0.679 |
| Q5: ≥5.03 | 285 (22.32) | 1.141 (0.940, 1.385) | 0.183 | 1.175 (0.966, 1.430) | 0.107 | 1.217 (0.991, 1.493) | 0.061 |
| p for trend | — | 0.070 | — | 0.035 | — | 0.017 | — |
| **TG, mmol/L** |  |  |  |  |  |  |  |
| Per SD increase | — | 1.025 (0.970, 1.084) | 0.384 | 1.013 (0.957, 1.073) | 0.650 | 1.006 (0.946, 1.070) | 0.843 |
| Q1: <1.06 | 238 (18.64) | 1.0 (Ref.) | — | 1.0 (Ref.) | — | 1.0 (Ref.) | — |
| Q2: 1.06≤TG<1.37 | 241 (18.87) | 0.965 (0.789, 1.179) | 0.727 | 0.951 (0.778, 1.164) | 0.628 | 0.952 (0.772, 1.175) | 0.650 |
| Q3: 1.37≤TG<1.71 | 260 (20.36) | 1.030 (0.845, 1.255) | 0.769 | 1.011 (0.828, 1.234) | 0.914 | 0.974 (0.790, 1.200) | 0.803 |
| Q4: 1.71≤TG<2.28 | 265 (20.75) | 1.035 (0.850, 1.260) | 0.734 | 1.010 (0.827, 1.232) | 0.925 | 0.932 (0.756, 1149) | 0.510 |
| Q5: ≥2.28 | 273 (21.38) | 1.104 (0.908, 1.344) | 0.321 | 1.062 (0.869, 1.299) | 0.555 | 0.995 (0.805, 1.232) | 0.966 |
| p for trend | — | 0.224 | — | 0.425 | — | 0.753 | — |
| **LDL-c, mmol/L** |  |  |  |  |  |  |  |
| Per SD increase | — | 1.146 (1.073, 1.225) | <0.001 | 1.151 (1.077, 1.231) | <0.001 | 1.159 (1.080, 1.243) | <0.001 |
| Q1: <1.76 | 222 (17.38) | 1.0 (Ref.) | — | 1.0 (Ref.) | — | 1.0 (Ref.) | — |
| Q2: 1.76≤LDL-c<2.16 | 237 (18.56) | 1.064 (0.868, 1.305) | 0.551 | 1.066 (0.869, 1.308) | 0.538 | 1.046 (0.846, 1.294) | 0..675 |
| Q3: 2.16≤LDL-c<2.59 | 242 (18.95) | 1.082 (0.883, 1.326) | 0.448 | 1.090 (0.889, 1.336) | 0.409 | 1.050 (0.850, 1.297) | 0.654 |
| Q4: 2.59≤LDL-c<3.19 | 277 (21.69) | 1.177 (0.966, 1.435) | 0.106 | 1.190 (0.976, 1.452) | 0.085 | 1.123 (0.913, 1.381) | 0.271 |
| Q5: ≥3.19 | 299 (23.41) | 1.343 (1.104, 1.633) | 0.003 | 1.362 (1.118, 1.658) | 0.002 | 1.354 (1.103, 1.663) | 0.004 |
| p for trend | — | 0.002 | — | 0.001 | — | <0.001 | — |
| **Non-HDL-c, mmol/L** |  |  |  |  |  |  |  |
| Per SD increase | — | 1.144 (1.078, 1.213) | <0.001 | 1.145 (1.079, 1.215) | <0.001 | 1.137 (1.067, 1.211) | <0.001 |
| Q1: <2.30 | 212 (16.60) | 1.0 (Ref.) | — | 1.0 (Ref.) | — | 1.0 (Ref.) | — |
| Q2: 2.30≤nHDL-c<2.78 | 246 (19.26) | 1.157 (0.942, 1.420) | 0.164 | 1.148 (0.935, 1.410) | 0.187 | 1.150 (0.929, 1.423) | 0.199 |
| Q3: 2.78≤nHDL-c<3.28 | 240 (18.79) | 1.032 (0.841, 1.268) | 0.761 | 1.033 (0.841, 1.269) | 0.758 | 0.951 (0.768, 1.179) | 0.649 |
| Q4: 3.28≤nHDL-c<3.96 | 273 (21.38) | 1.232 (1.008, 1.506) | 0.042 | 1.230 (1.005, 1.505) | 0.045 | 1.143 (0.926, 1.412) | 0.213 |
| Q5: ≥3.96 | 306 (23.96) | 1.443 (1.185, 1.759) | <0.001 | 1.448 (1.186, 1.768) | <0.001 | 1.399 (1.136, 1.724) | 0.002 |
| p for trend | — | <0.001 | — | <0.001 | — | <0.001 | — |
| **Lp(a), mg/dL** |  |  |  |  |  |  |  |
| Per SD increase | — | 1.003 (1.001, 1.005) | 0.008 | 1.003 (1.001, 1.005) | 0.003 | 1.002 (1.000, 1.005) | 0.032 |
| Q1: <6.27 | 213 (16.68) | 1.0 (Ref.) | — | 1.0 (Ref.) | — | 1.0 (Ref.) | — |
| Q2: 6.27≤lp(a)<13.08 | 242 (18.95) | 1.143 (0.932, 1.403) | 0.200 | 1.159 (0.944, 1.422) | 0.159 | 1.084 (0.876, 1.342) | 0.457 |
| Q3: 13.08≤lp(a)<25.21 | 283 (22.16) | 1.338 (1.097, 1.633) | 0.004 | 1.358 (1.113, 1.658) | 0.003 | 1.308 (1.063, 1.608) | 0.011 |
| Q4: 25.21≤lp(a)<48.42 | 259 (20.28) | 1.267 (1.035, 1.552) | 0.022 | 1.289 (1.052, 1.579) | 0.014 | 1.159 (0.939, 1.432) | 0.170 |
| Q5: ≥48.42 | 280 (21.93) | 1.348 (1.104, 1.646) | 0.004 | 1.395 (1.141, 1.705) | 0.001 | 1.271 (1.032, 1.566) | 0.024 |
| p for trend | — | 0.002 | — | 0.001 | — | <0.001 | — |
| **ApoB, g/L** |  |  |  |  |  |  |  |
| Per SD increase | — | 1.694 (1.331, 2.157) | <0.001 | 1.693 (1.326, 2.160) | <0.001 | 1.623 (1.255, 2.099) | <0.001 |
| Q1: <0.63 | 223 (17.46) | 1.0 (Ref.) | — | 1.0 (Ref.) | — | 1.0 (Ref.) | — |
| Q2: 0.63≤apoB<0.75 | 231 (18.09) | 1.044 (0.850, 1.281) | 0.683 | 1.035 (0.842, 1.271) | 0.745 | 0.971 (0.784, 1.202) | 0.786 |
| Q3: 0.75≤apoB<0.86 | 224 (17.54) | 1.010 (0.822, 1.241) | 0.926 | 1.005 (0.817, 1.236) | 0.965 | 0.968 (0.781, 1.200) | 0.766 |
| Q4: 0.86≤apoB<1.02 | 286 (22.40) | 1.164 (0.957, 1.417) | 0.128 | 1.163 (0.955, 1.417) | 0.132 | 1.081 (0.880, 1.328) | 0.460 |
| Q5: ≥1.02 | 313 (24.51) | 1.440 (1.186, 1.750) | <0.001 | 1.436 (1.180, 1.747) | <0.001 | 1.333 (1.085, 1.636) | 0.006 |
| p for trend | — | <0.001 | — | <0.001 | — | <0.001 | — |
| **Non-HDL-c/HDL-c** |  |  |  |  |  |  |  |
| Per SD increase | — | 1.180 (1.131, 1.230) | <0.001 | 1.170 (1.121, 1.221) | <0.001 | 1.118 (1.068, 1.170) | <0.001 |
| Q1: <2.17 | 187 (14.64) | 1.0 (Ref.) | — | 1.0 (Ref.) | — | 1.0 (Ref.) | — |
| Q2: 2.17≤ratio<2.74 | 191 (14.96) | 1.002 (0.804, 1.250) | 0.985 | 0.989 (0.793, 1.234) | 0.923 | 0.989 (0.791, 1.236) | 0.920 |
| Q3: 2.74≤ratio<3.39 | 265 (20.75) | 1.437 (1.167, 1.770) | 0.001 | 1.412 (1.146, 1.740) | 0.001 | 1.415 (1.145, 1.748) | 0.001 |
| Q4: 3.39≤ratio<4.27 | 274 (21.46) | 1.398(1.137, 1.718) | 0.001 | 1.367 (1.111, 1.683) | 0.003 | 1.380 (1.118, 1.703) | 0.002 |
| Q5: ≥4.27 | 360 (28.19) | 1.897 (1.555, 2.313) | <0.001 | 1.826 (1.491, 2.235) | <0.001 | 1.814 (1.475, 2.231) | <0.001 |
| p for trend | — | <0.001 | — | <0.001 | — | <0.001 | — |
| **ApoB/apoA-1** |  |  |  |  |  |  |  |
| Per SD increase | — | 3.192 (2.428, 4.195) | <0.001 | 3.030 (2.292, 4.005) | <0.001 | 2.239 (1.665, 3.011) | <0.001 |
| Q1: <0.47 | 178 (13.94) | 1.0 (Ref.) | — | 1.0 (Ref.) | — | 1.0 (Ref.) | — |
| Q2: 0.47≤ratio<0.57 | 207 (16.21) | 1.193 (0.958, 1.486) | 0.116 | 1.169 (0.938, 1.457) | 0.165 | 1.165 (0.933, 1.455) | 0.177 |
| Q3: 0.57≤ratio<0.66 | 227 (17.78) | 1.259 (1.015, 1.563) | 0.036 | 1.232 (1.012, 1.530) | 0.046 | 1.232 (1.000, 1.533) | 0.050 |
| Q4: 0.66≤ratio<0.79 | 284 (22.24) | 1.577 (1.281, 1.941) | <0.001 | 1.535 (1.245, 1.893) | <0.001 | 1.543 (1.249, 1.906) | <0.001 |
| Q5: ≥0.79 | 381 (29.84) | 2.175 (1.781, 2.655) | <0.001 | 2.097 (1.713, 2.568) | <0.001 | 2.115 (1.722, 2.597) | <0.001 |
| p for trend | — | <0.001 | — | <0.001 | — | <0.001 | — |

Model 1 was the crude model. Model 2 was adjusted for sex and age. Model 3 was adjusted for sex, age, body mass index, hypertension, diabetes, prior myocardial infarction, prior percutaneous coronary intervention, and smoking history.

OR, odds ratio; CI, confidence interval; SD, standard deviation; Q, quintile; Ref., reference.

# Supplemental Table12. Predictive value of different lipid measures for total occlusion in patients with acute coronary syndrome.

|  | **AUROC (95% CI)** | **P for comparison** | **IDI** | **p** |
| --- | --- | --- | --- | --- |
| **Total cholesterol** | 0.517 (0.499, 0.536) | — | Reference | — |
| **Total triglyceride** | 0.508 (0.490, 0.525) | 0.349 | -0.017 | 0.328 |
| **LDL-c** | 0.532 (0.514, 0.550) | <0.001 | 0.014 | 0.312 |
| **Non-HDL-c** | 0.537 (0.519, 0.555) | <0.001 | 0.020 | 0.044 |
| **Lp(a)** | 0.528 (0.510, 0.545) | 0.387 | 0.016 | 0.055 |
| **ApoB** | 0.535 (0.517, 0.553) | 0.001 | 0.024 | 0.046 |
| **Non-HDL-c/HDL-c** | 0.569 (0.551, 0.586) | <0.001 | 0.069 | <0.001 |
| **ApoB/apoA-1** | 0.577 (0.559, 0.595) | <0.001 | 0.093 | <0.001 |

AUROC, area under the receiver operating characteristic curve; CI, confidence interval; IDI, integrated discrimination index; apo, apolipoprotein; LDL-c, low-density lipoprotein cholesterol; HDL-c, high-density lipoprotein cholesterol; lp(a), lipoprotein (a).

# Supplemental Table13. Discordance analysis between apoB/apoA-1 and non-HDL-c/HDL-c in patients with acute coronary syndrome.

| **Non-HDL-c/HDL-c** | **ApoB/apoA-1** | | | | | |
| --- | --- | --- | --- | --- | --- | --- |
|  | **Low** | | **Middle** | | **High** | |
|  | **Number (%)** | **OR (95% CI)** | **Number (%)** | **OR (95% CI)** | **Number (%)** | **OR (95% CI)** |
| **Low** | 221 (15.84) | 1.0 (Ref.) | 85 (18.93) | 1.236 (0.936, 1.633) | 9 (25.00) | 1.787 (0.824, 3.874) |
| **Middle** | 86 (18.86) | 1.209 (0.916, 1.597) | 205 (19.98) | 1.317 (1.065, 1.630) | 126 (25.20) | 1.777 (1.382, 2.284) |
| **High** | 10 (20.83) | 1.345 (0.964, 1.397) | 112 (22.00) | 1.473 (1.082, 2.456) | 423 (26.40) | 1.872 (1.644, 2.389) |

Adjusted for sex, age, body mass index, hypertension, diabetes, prior myocardial infarction, prior percutaneous coronary intervention, and smoking history. Apo, apolipoprotein; LDL-c, low-density lipoprotein cholesterol; HDL-c, high-density lipoprotein cholesterol; OR, odds ratio; CI, confidence interval; Ref., reference.

# Supplemental Figure1. Distribution of each lipid measure. LDL-c, low-density lipoprotein cholesterol; HDL-c, high-density lipoprotein cholesterol; Lp(a), lipoprotein (a); apo, apolipoprotein.


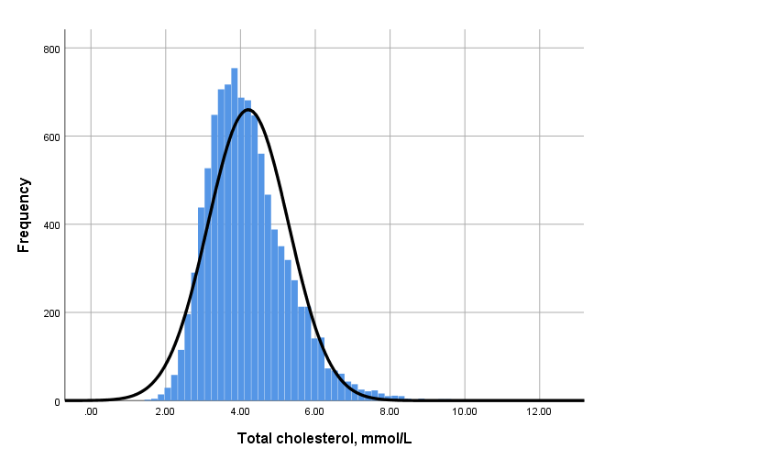

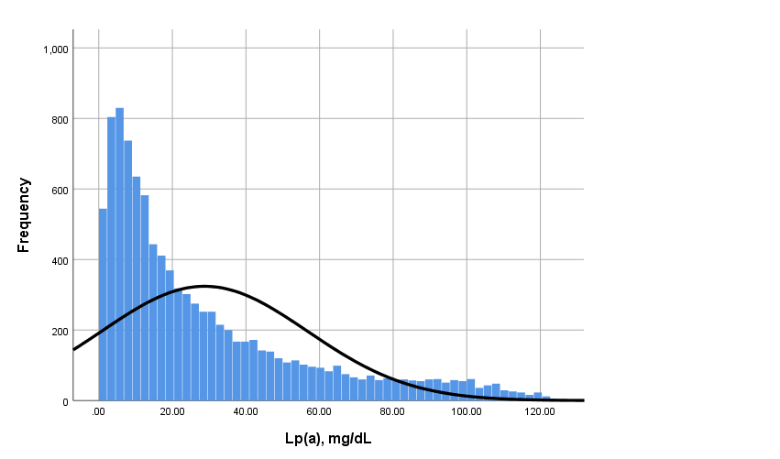

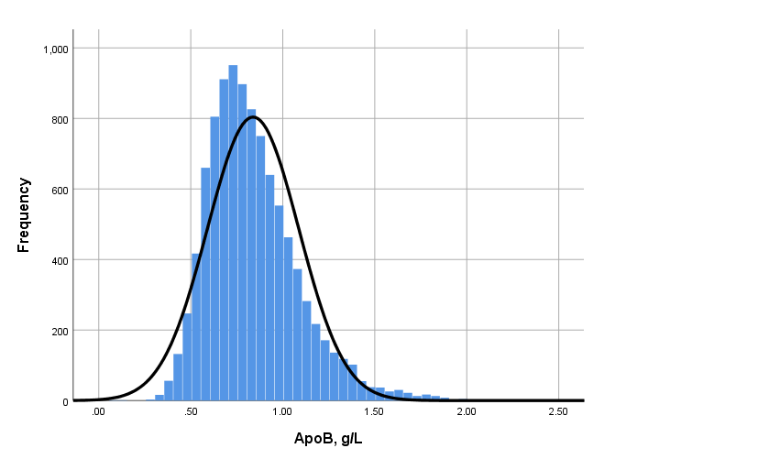

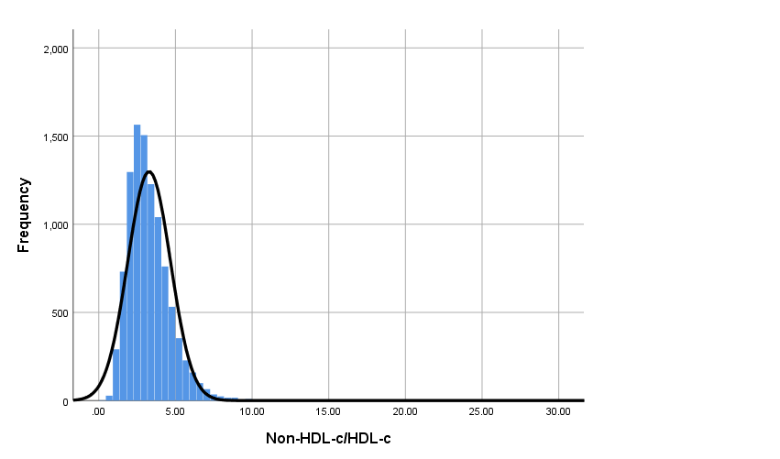

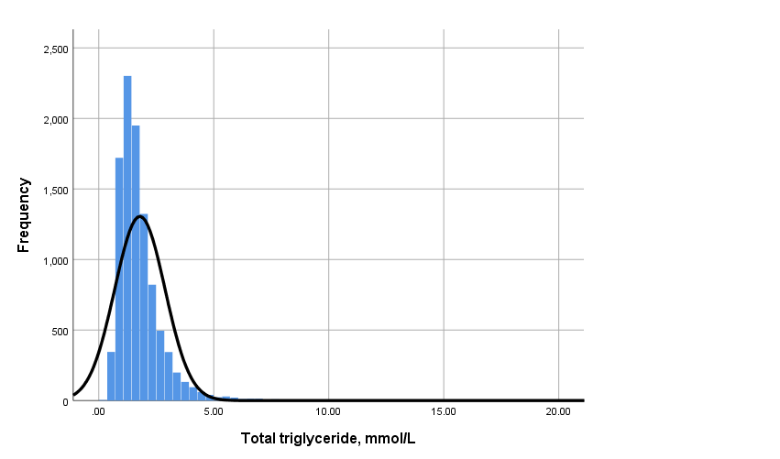

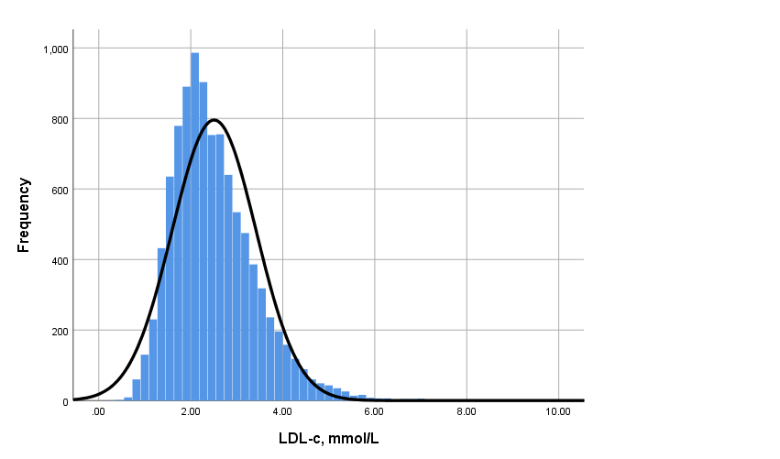

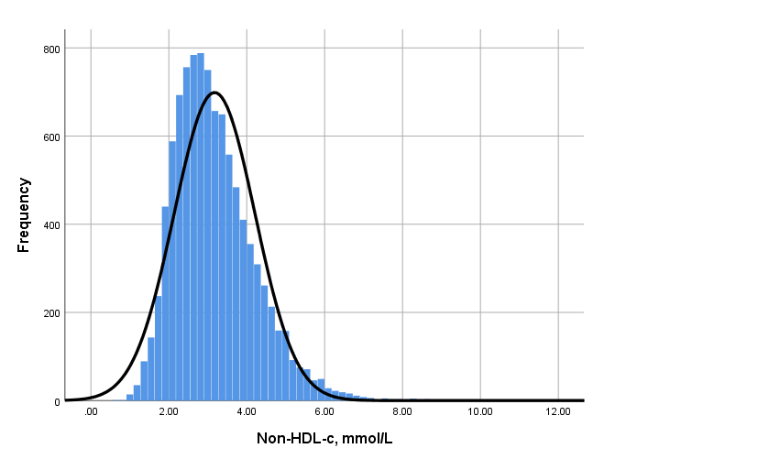

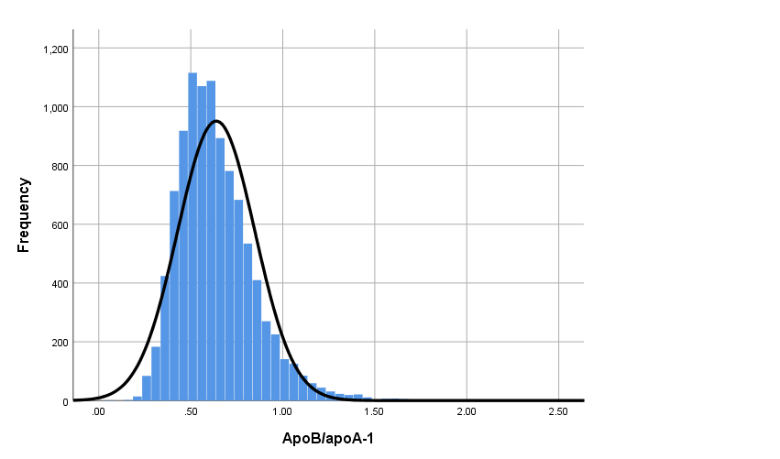


# Supplemental Reference

[1] easyROC: a web-tool for ROC curve analysis (ver. 1.3.1). 2022-09-23. http://www.biosoft.hacettepe.edu.tr/easyROC/

[2] Goksuluk D, Korkmaz S, Zararsiz G, Karaağaoğlu AE (2016). easyROC: An Interactive Web-tool for ROC Curve Analysis Using R Language Environment. The R Journal, 8(2):213-230.
